# KRAS-Driven Hypertranscription and Metastatic Dissemination in Colorectal Cancer Could be Overcome by Targeting the NMHC IIA/ PLK1 Signaling Axis with a Novel Acridine Derivative

**DOI:** 10.7150/ijbs.121816

**Published:** 2026-01-01

**Authors:** Dengbo Ji, Haizhao Yi, Ming Li, Zhaoya Gao, Rui Yang, Jingying Jia, Xinxin Cui, Can Song, Hanyang Wang, Mengyuan Shi, Yuhao Yan, Tongtong Geng, Xiangbao Meng, Zhongjun Li, Jin Gu

**Affiliations:** 1 Key laboratory of Carcinogenesis and Translational Research (Ministry of Education), Department of Gastrointestinal Surgery III, Peking University Cancer Hospital & Institute, No. 52 Fucheng Rd., Haidian District, Beijing, China, 100142.; 2 Peking University S.G. Hospital, Beijing, China.; 3 The State Key Laboratory of Natural and Biomimetic Drugs, Peking University, Beijing, China, 100191.; 4 School of Life Sciences, Tsinghua University, Beijing, China, 100084.; 5 Peking-Tsinghua Center for Life Sciences, Beijing, China.; 6 Department of General Surgery, Affiliated Hospital of Chengde Medical College, Chengde, China.

**Keywords:** KRAS-mutated colorectal cancer, NMHC IIA, YAP/FOXO/PLK1 pathway, acridine derivative.

## Abstract

Colorectal cancer (CRC) becomes highly lethal upon progression to advanced or metastatic stages. Treatment options are particularly limited for refractory metastatic CRC (mCRC) harboring KRAS mutations. In this study, we established a series of patient-derived organoids (PDOs) and patient-derived xenografts (PDXs) from mCRC patients to identify effective therapeutic compounds. We employed RNA sequencing to characterize the transcriptomic profiles of KRAS-mutant microsatellite stable (MSS) PDOs and analyzed single-cell RNA sequencing data to examine features of KRAS-mutant CRC epithelial cells. Transcriptomic analysis revealed that KRAS mutations induce elevated global transcription activity in both PDOs and epithelial cells. A large-scale drug screen of 786 Food and Drug Administration (FDA)-approved anticancer agents identified the acridine compound amsacrine hydrochloride as a potent inhibitor of PDOs and cell lines. We subsequently synthesized a series of acridine derivatives for further screening. Finally, LS-1-2 was discovered to overcome chemotherapy resistance and suppress liver metastasis in KRAS-mutated CRC. Mechanistically, LS-1-2 binds to non-muscle myosin heavy chain IIA (NMHC IIA), blocking its phosphorylation. This inhibition disrupts the PI3K/ERK/FOXO/PLK1 signaling pathway and attenuates KRAS-driven hypertranscription. In conclusion, the acridine derivative LS-1-2 emerges as a promising candidate from this preclinical investigation, providing a rationale for future clinical trials in KRAS-mutant CRC.

## Introduction

KRAS mutations are present in approximately 45% of colorectal cancer (CRC) patients. Studies have indicated that patients with KRAS-mutated metastatic CRC (mCRC) exhibit worse overall and progression-free survival compared to those with wild-type KRAS 1. Recent progress has been made in developing drugs that target KRAS, particularly with the discovery of agents directed against the G12C mutation 2. However, the prevalence of the KRAS G12C mutation in CRC is very low 3. Consequently, discovering effective treatments for KRAS-mutated mCRC remains a critical unmet need.

In this study, transcriptomic profiling revealed that KRAS mutations drive increased global transcription activity. We established drug screening strategies to identify inhibitors for KRAS-mutated mCRC. Specifically, we generated a series of PDOs and PDXs from KRAS-mutated metastatic colorectal cancers, with an emphasis on refractory cases, to identify the most effective therapeutic compounds. A high-throughput screen of an FDA-approved oncology drug library identified the acridine derivative amsacrine hydrochloride (m-AMSA) as a selective inhibitor of KRAS-mutant PDOs and cell lines. Although m-AMSA shows clinical activity in hematologic malignancies such as acute leukemia and lymphoma 4, 5, its limited efficacy against solid tumors is likely due to pharmacokinetic barriers or microenvironmental resistance 6, 7. To address these limitations, we designed a structure-guided library of pyrrolo[2,3,4-*kl*]acridin-1(2*H*)-one derivatives, employing scaffold hopping strategies to enhance solid tumor penetration and tolerability. From this series, LS-1-2 emerged as a preclinical drug candidate, demonstrating potent anti-proliferative and anti-metastatic efficacy in both *in vitro* and *in vivo* models of KRAS-mutant colorectal cancer. By integrating biochemical assays with multi-omics analyses, including phosphoproteomics and RNA-seq, we systematically investigated the mechanism of action of LS-1-2 and identified its targeting of the NMHC IIA/FOXO/PLK1 signaling pathway.

## Materials and Methods

### Patients and Samples

CRC tissue samples utilized in this study were obtained from Peking University Shougang Hospital. The Ethics Review Committees of Peking University Shougang Hospital approved the collection and use of these samples in accordance with the Declaration of Helsinki (IRBK-2021-026-01, 2021.7). The clinical characteristics of these patients are summarized in Tables S1-3 and Figure S1.

### Patient-Derived Organoid Culture and Drug Assay

Patient-derived organoids were cultured according to methods described in a previous study 8. Detailed culture protocols are provided in the supplemental materials. For drug assays, organoids in good condition were harvested, passaged, and seeded into 96-well cell culture plates. The standard organoid culture medium was replaced with drug-containing medium. Organoid cell viability was assessed using the CellTiter-Glo 3D Cell Viability Assay (Promega, G9683).

### Patient-Derived Cell Line (PDC) Establishment and Culture

Tumors established from PDXs were excised and processed for *in vitro* culture. Detailed culture methods are provided in the supplemental materials.

### Cell Lines

Human colorectal cancer cell lines (CL11, CL40, LS513, HCT116, HCT8, SW480, SW620, LOVO, and LS174T; all KRAS mutant) were obtained from the American Type Culture Collection (USA) and cultured in DMEM (Gibco) supplemented with 10% fetal bovine serum at 37°C under 5% CO₂. The DiFi cell line (KRAS wild-type) was acquired from Nanjing Cobioer Biosciences. Chemoresistant HCT8-5Fu cells were generated through gradual exposure to 5-fluorouracil. HCT116/self cells were established from stem cell-enriched spheres as previously described 9. All cell lines were authenticated and verified using short tandem repeat (STR) profiling. KRAS mutation status of the CRC cells used in this manuscript in Table S4.

### Drug Library Construction and Screening Strategy

To identify potential therapeutic agents for KRAS-mutant microsatellite stable (MSS) colorectal cancer, we constructed a curated drug library by integrating compounds from two established repositories: the Anti-cancer Compound Library (L3000, Selleck) and the FDA-approved Drug Library (L1300, Selleck). This integrated approach yielded a focused collection of 786 clinically relevant compounds for evaluation. All included drugs meet two critical criteria: documented biological activity and established safety profiles from prior clinical trials, and broad coverage of signaling pathways and molecular targets implicated in oncogenesis and therapeutic resistance. These compounds target diverse tumor-related mechanisms, including key signaling pathways such as mitogen-activated protein kinase (MAPK), Wnt, PI3K/protein kinase B (Akt)/mammalian target of rapamycin (mTOR), Janus kinase/signal transducer and activator of transcription (JAK/STAT), and nuclear factor-kappa B (NF-κB). They also target fundamental cellular processes including apoptosis, angiogenesis, autophagy, cell cycle regulation, DNA damage response, and cytoskeletal signaling, in addition to specific targets such as protein tyrosine kinases, G protein-coupled receptors, and the proteasome.

We additionally synthesized 131 small molecules featuring a pyrrolo[2,3,4-kl]acridin-1(2H)-one scaffold as an internal compound library for further screening.

### RNA Sequencing and RNA Microarray Analysis in Organoids and Cell Lines

RNA sequencing of PDOs samples was performed on an Illumina platform by Annoroad Gene Technology. The RNA microarray analysis for cell lines was conducted by Shanghai Biotechnology Corporation. Detailed experimental protocols are provided in the supplemental materials.

### Immunofluorescent Staining and Flow Cytometry

Cells were isolated either from culture using 0.02% EDTA/PBS or through mechanical homogenization of primary or xenografted tumors. The isolated cells were stained with a mouse anti-CD133 monoclonal antibody (1:11 dilution, Miltenyi Biotec, 130-098-046). Analysis of labeled samples was performed using a FACSAria II flow cytometer (BD Biosciences, San Jose, CA).

### *In vivo* Mouse Studies

Male BALB/c-nu and NOD-SCID mice (4-6 weeks old) were obtained from Beijing HFK Bio-technology Co., Ltd., and maintained under pathogen-free conditions in accordance with institutional animal care protocols (IACUC, Peking University).

The activity of LS-1-2 was evaluated *in vivo* using KRAS-mutated colorectal cancer cell line-derived xenograft (CDX) models and PDX models. CDX models were established by subcutaneously injecting 5×10⁵ tumor cells into NOD/SCID mice. PDX models were generated as previously described 10 and included PDX1 (colon cancer), PDX2 and PDX3 (primary and liver metastatic lesions from a colon cancer patient), and PDX4 (rectal cancer after neoadjuvant chemoradiotherapy: 50.6 Gy/22 fractions with concurrent capecitabine [825 mg/m² twice daily for 5 weeks]). When tumor volumes reached 100-200 mm³, mice were randomized into treatment or control groups (3-5 mice per group).

For liver metastasis models, LS174T-luciferin cells (1×10⁵) were injected into the splenic subcapsule of nude mice. Treatment regimens included LS-1-2 (10, 20, or 40 mg/kg, intraperitoneally, days 1-5 weekly), 5-fluorouracil (5-FU, 30 mg/kg/week), irinotecan (CPT-11, 20 mg/kg/week), or TAS-102 (150 mg/kg twice daily, orally). Control animals received vehicle (PBS). TAS-102 (trifluridine-tipiracil) is an orally administered, well-tolerated combination drug composed of trifluridine, a thymidine analogue, and tipiracil, a thymidine phosphorylase inhibitor. Tumor volume was calculated as 0.52 × length × width × height and measured twice weekly. Metastatic burden was quantified using IVIS Spectrum-CT bioluminescence imaging (PerkinElmer) and analyzed with Living Image 4.0 software (Caliper Life Sciences).

*In vivo* studies were terminated either upon animal death or when the tumor burden reached a protocol-specified maximum dimension of 1.5 cm, as recommended in prior guidelines.

### Quantitative Phosphoproteomic Analysis

Quantitative phosphoproteomic analysis was performed according to the standard procedures of Jingjie PTM BioLab (Hangzhou, China). Detailed experimental methods are provided in the supplemental materials. To identify high-confidence phosphopeptides regulated by LS-1-2, we applied filtering criteria of a fold change greater than 1.3 or less than 0.77 with a P value < 0.05 (Student's t-test). A global protein interaction network for the significantly altered phosphoproteins was constructed using the STRING database and annotated with KEGG pathways. To elucidate the signaling pathway affected by LS-1-2 through its interaction with MYH9, we analyzed interactions between changed phosphoproteins, differentially expressed mRNAs, and MYH9 protein using the STRING database, with pathway enrichment determined by KEGG annotation.

Kinase-substrate relationships were predicted using iGPS1.0 software, which operates on the principle that short linear motifs surrounding phosphorylation sites determine primary specificity. The software employed the GPS2.0 algorithm 11 to predict site-specific kinase-substrate relations, with protein-protein interaction data used as a major contextual filter to reduce false-positive predictions. A "medium" threshold was selected with the "Interaction" parameter set to "Exp./String." Kinase activities were predicted using Gene Set Enrichment Analysis (GSEA), with log-transformed phosphorylation levels served as the ranking metric and kinase-phosphorylation site regulations were formatted into a .gmt file. Normalized enrichment scores from the GSEA results were interpreted as kinase activity scores. A kinase was predicted as activated if its substrates showed predominantly increased phosphorylation, and inhibited if the opposite pattern was observed. Analysis of the Clinical Proteomic Tumor Analysis Consortium (CPTAC) data was conducted through the UALCAN platform (https://ualcan.path.uab.edu/analysis-prot.html) to examine MYH9 protein expression and phosphorylation levels in colorectal cancer and their association with clinical stages.

### Biotin Pull-Down Assay and Protein Identification by Liquid Chromatography-Tandem Mass Spectrometry (LC-MS/MS)

HCT116 cell lysates were incubated with biotin, biotin-tagged LS-1-2 (LS-1-2-Biotin), or LS-1-2-Biotin in the presence of excess untagged LS-1-2 at 4 °C for 4 h. Protein complexes were isolated using the Pierce™ Pull-Down Biotinylated Protein: Protein Interaction Kit (Thermo Scientific) according to the manufacturer's protocol. Samples were separated by sodium dodecyl sulfate-polyacrylamide gel electrophoresis (SDS-PAGE) and visualized by silver staining. Specific protein bands were excised, subjected to in-gel tryptic digestion, and identified by LC-MS/MS analysis.

### Molecular Docking and Molecular Dynamics

The amino acid sequence of non-muscle myosin heavy chain IIA (NMHC IIA; Accession No.: ALX00019.1) was retrieved from the National Center for Biotechnology Information protein database. The three-dimensional structure of NMHC IIA was generated using Rosetta 8. Detailed methodological descriptions are provided in the supplementary materials.

### ATPase activity assay

ATPase activity was measured using an optimized molybdenum blue method 12. Recombinant NMHC IIA protein (ab226278, Abcam) was pre-incubated with either Blebbistatin or LS-1-2 for 10 minutes, while the control group received 0.1% DMSO. Following the enzymatic reaction, ammonium molybdate was added to quantify released inorganic phosphate.

### Cellular Thermal Shift Assay (CETSA)

Detailed procedures for the cellular thermal shift assay are provided in the supplementary materials.

### Statistical Analysis

Data are presented as mean ±SE and were analyzed using SPSS 19.0. Associations between marker expression and clinicopathological characteristics were evaluated using Spearman's correlation analysis. Survival curves were constructed by Kaplan-Meier methodology and compared using log-rank tests. Data from *in vitro* and *in vivo* experiments were analyzed by unpaired two-tailed t-tests or one-way ANOVA with Tukey's post hoc test. All *in vitro* experiments were independently repeated at least three times, and group sizes for animal studies are indicated in the respective figures. Randomization was implemented when tumors reached predetermined volumes.

## Results

### Drug Screening Identifies Acridine-Based Therapeutics for KRAS-Mutant MSS CRC

**Figure 1:**
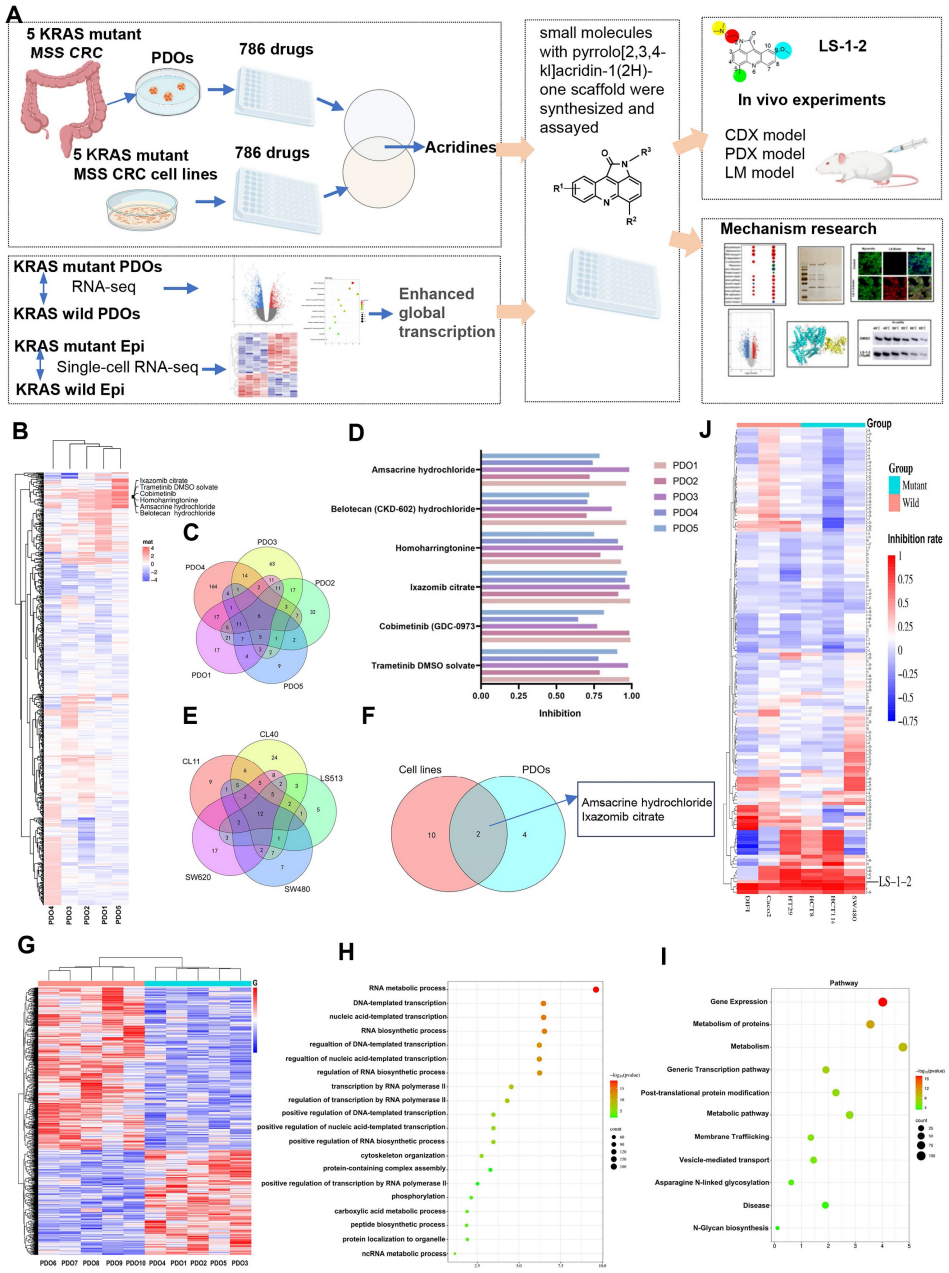
** Comprehensive Drug Screening and Transcriptomic Profiling in KRAS-Mutant MSS CRC. A.** Schematic overview of the integrated drug discovery and molecular characterization pipeline. **B.** Drug response heatmap for five KRAS-mutant MSS CRC patient-derived organoids (PDOs) screened against the FDA-approved Drug Library. **C.** Venn diagram identifying six compounds with >60% growth inhibition across all five PDOs. **D.** Comparative inhibition rates of shared candidate drugs in individual PDO models. **E.** Venn analysis revealing twelve agents with >60% efficacy in five KRAS-mutant MSS CRC cell lines. **F.** Intersection analysis of PDO- and cell line-active compounds, highlighting overlapping therapeutic candidates. **G.** Transcriptomic heatmap contrasting gene expression profiles between KRAS-mutant and KRAS wild-type MSS CRC PDOs. **H.** Gene Ontology (GO) enrichment analysis showing top 20 upregulated biological processes in KRAS-mutant PDOs. **I.** KEGG pathway analysis identifying eleven significantly activated signaling cascades in KRAS-mutant MSS CRC PDOs. **J.** Heatmap of drug inhibition for KRAS-mutant and KRAS wild-type cell lines using an internal compound library.

To address the therapeutic challenges in KRAS-mutant MSS CRC, we implemented a dual-platform screening strategy utilizing five PDOs and five cell line models (Figure 1A). All PDOs were derived from patients with advanced KRAS-mutant MSS CRC who demonstrated limited clinical benefit from prior multidrug therapies, thereby reflecting real-world treatment resistance. Representative patient treatment histories are documented in Figure S1.

From the 786 compounds screened in the FDA-approved Drug Library, six agents targeting MEK, proteasome, protein synthesis, and DNA damage pathways achieved more than 60% growth inhibition across all five PDOs (Figure 1B-D). Twelve compounds directed against proteasome complexes, DNA repair machinery, and histone deacetylase (HDAC) demonstrated greater than 60% efficacy in all cell lines (Figure 1E). The acridine derivative amsacrine hydrochloride and the proteasome inhibitor ixazomib citrate showed consistent and potent antitumor activity in both biological systems (Figure 1F).

### Enhanced Global Transcription Activity in KRAS-Mutant CRC Cells

To delineate molecular distinctions between KRAS-mutant and KRAS wild-type MSS CRC, we performed RNA sequencing on five KRAS-mutant and five KRAS wild-type PDOs. Functional genomics analyses, including Gene Ontology and KEGG pathway enrichment, revealed pronounced activation of transcriptional regulatory networks in KRAS-mutant MSS PDOs. Upregulated genes were significantly enriched in pathways governing RNA metabolic processes, RNA biosynthetic processes, and regulation of DNA-templated transcription (Figure 1G-I). Gene Ontology biological process analysis demonstrated that significantly elevated genes in KRAS-mutant PDOs were primarily associated with RNA metabolism, DNA-templated transcription, and regulation of transcription by RNA polymerase II. DNA-templated transcription and RNA polymerase II-driven transcription regulation represent key steps in gene expression, encompassing mRNA synthesis and the production of certain small nuclear RNAs. KEGG pathway analysis identified Gene Expression as the most significantly enriched pathway. These results collectively indicate that KRAS mutation promotes a global upregulation of transcriptional activity.

We performed comparative transcriptomic analysis of KRAS-mutant versus wild-type CRC epithelial cells using single-cell RNA sequencing data from a published cohort 13, in which mutational status was validated through targeted cDNA Sanger sequencing at single-cell resolution. Consistent with our PDO findings, differential gene expression analysis revealed similar enrichment patterns in RNA transcription-related biological processes and associated regulatory pathways (Figure S2). This concordance between organoid models and primary tumor cells underscores the robustness of transcriptional network activation as a hallmark feature of KRAS-mutant MSS CRC.

### Discovery of LS-1-2

**Figure 2:**
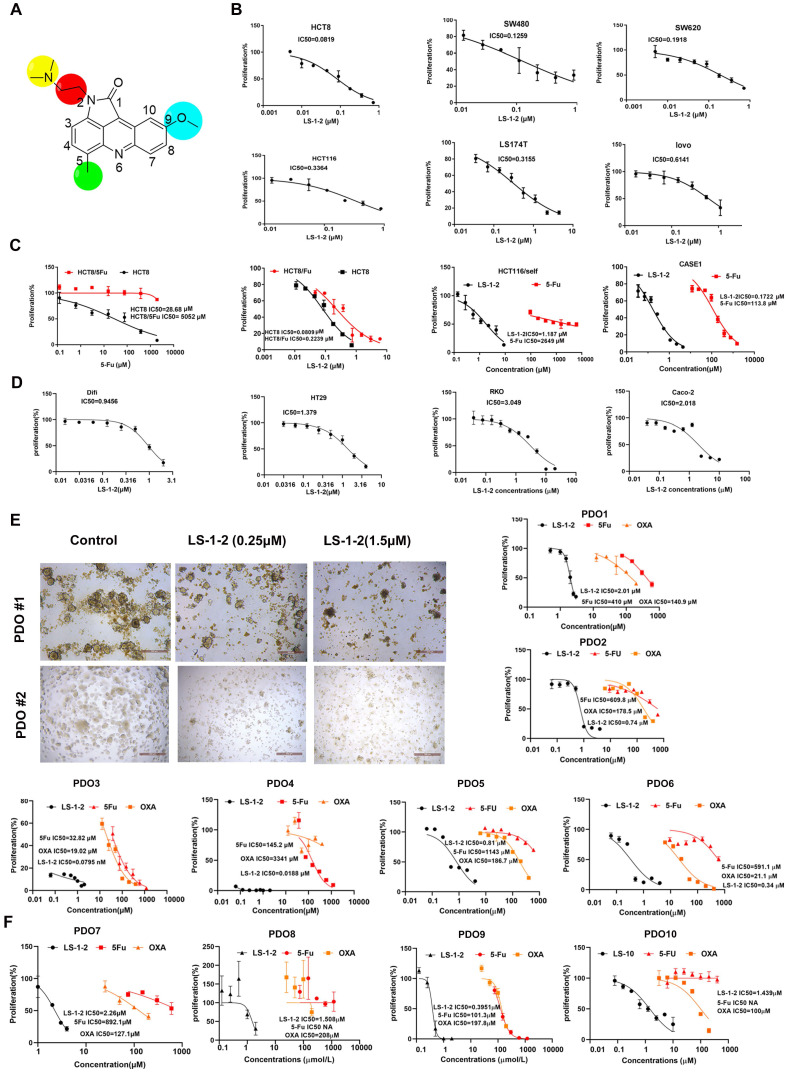
** LS-1-2 exhibits broad antiproliferative effects on CRC**. **A.** Chemical structure of LS-1-2. **B.** Dose-response of the proliferation of KRAS mutant CRC cell lines exposed to LS-1-2 for 48 h. **C**. Dose-response of the proliferation of 5-FU resistant HCT8, HCT116/self cells, and PDC-CASE1 cells exposed to LS-1-2 or 5-FU for 48 h. **D**. Dose-response of the proliferation of KRAS wild type CRC cell lines exposed to LS-1-2 for 48 h. **E**. Left; Representative photomicrographs of CRC PDOs (PDO #1-2) treated with DMSO or with 0.25 or 1.5 μM LS-1-2 (40× magnification). Right: CellTiter-Glo measured the cell viability of KRAS mutant CRC PDOs (1-6). **F**. The cell viability of KRAS wild type CRC PDOs (7-10).

To identify inhibitors of KRAS-mutated CRC proliferation, we screened 131 pyrrolo[2,3,4-*kl*]acridin-1(2*H*)-one scaffold small molecules from our internal library against KRAS-mutant (HCT8, HCT116, SW480) and KRAS wild-type (HT29, Difi, Caco2) CRC cell lines (Figure 1J, Table S5). Three compounds demonstrated robust inhibitory effects on KRAS-mutant cell lines. Among these, LS-1-2 showed significantly greater potency against KRAS-mutant cells (inhibition rates: 93.8%, 86.7%, and 76.2%) compared to wild-type cells (inhibition rates: 83.7%, 35.4%, and 50%), while the other two compounds exhibited no statistically significant genotype selectivity (Figure 1J). LS-1-2 (Figure 2A) was subsequently evaluated in additional CRC cell lines and PDO models, confirming its broad antiproliferative efficacy against KRAS-mutated CRC cells.

LS-1-2 demonstrated dose-dependent antiproliferative activity against KRAS-mutant CRC cell lines (IC₅₀: 0.0819-0.9456 μM) and chemoresistant variants (HCT8-5-FU: 0.2239 μM; HCT116/self: 1.187 μM; PDC1: 0.1722 μM) (Figure 2B-C). While LS-1-2 also inhibited the growth of KRAS wild-type CRC cells, these cells exhibited reduced sensitivity (Difi IC₅₀ = 0.9456 μM, HT29 IC₅₀ = 1.379 μM, RKO IC₅₀ = 3.049 μM, Caco-2 IC₅₀ = 2.018 μM) compared to KRAS-mutant counterparts (Figure 2D).

Similar differential responses were observed in PDOs. Across six KRAS-mutant metastatic CRC PDOs, including paired primary and recurrent lesions from one patient, LS-1-2 suppressed growth in a dose-dependent manner despite substantial heterogeneity in baseline chemosensitivity to conventional agents (5-FU IC₅₀: 32.82-1143 μM; oxaliplatin IC₅₀: 19.02-3341 μM) (Figure 2E). Notably, PDOs derived from multidrug-refractory patients (previously treated with fluoropyrimidine/irinotecan/oxaliplatin with or without bevacizumab/radiotherapy; Figure S1) remained sensitive to LS-1-2, confirming its translational potential for treatment-resistant KRAS-mutant CRC. Although LS-1-2 also showed efficacy in KRAS wild-type PDOs (IC₅₀: 0.3951-2.26 μM), a moderate reduction in sensitivity was consistently observed relative to KRAS-mutant PDOs (Figure 2F).

### LS-1-2 Overcomes KRAS-Mutated CRC Growth and Resistance to 5-FU* In vivo*

The *in vivo* antitumor activity of LS-1-2 was evaluated using HCT116 and HCT8/5Fu cell-derived xenograft (CDX) models and four KRAS-mutated patient-derived xenograft (PDX1-4) models. Mice were treated for 18 days with PBS (control), LS-1-2 (10, 20, or 40 mg/kg), 5-FU (30 mg/kg), or CPT-11 (20 mg/kg). LS-1-2 significantly inhibited HCT116 tumor growth in a dose-dependent manner compared to the control (all p < 0.01; Figure S3A). At 40 mg/kg, LS-1-2 demonstrated superior tumor growth inhibition relative to 5-FU (p = 0.0414 for tumor weight; p = 0.0005 for tumor inhibition rate). In the 5-FU-resistant HCT8/5-FU model, LS-1-2 (40 mg/kg) also effectively suppressed tumor growth compared to both the control and 5-FU groups (Figure S3B).

**Figure 3:**
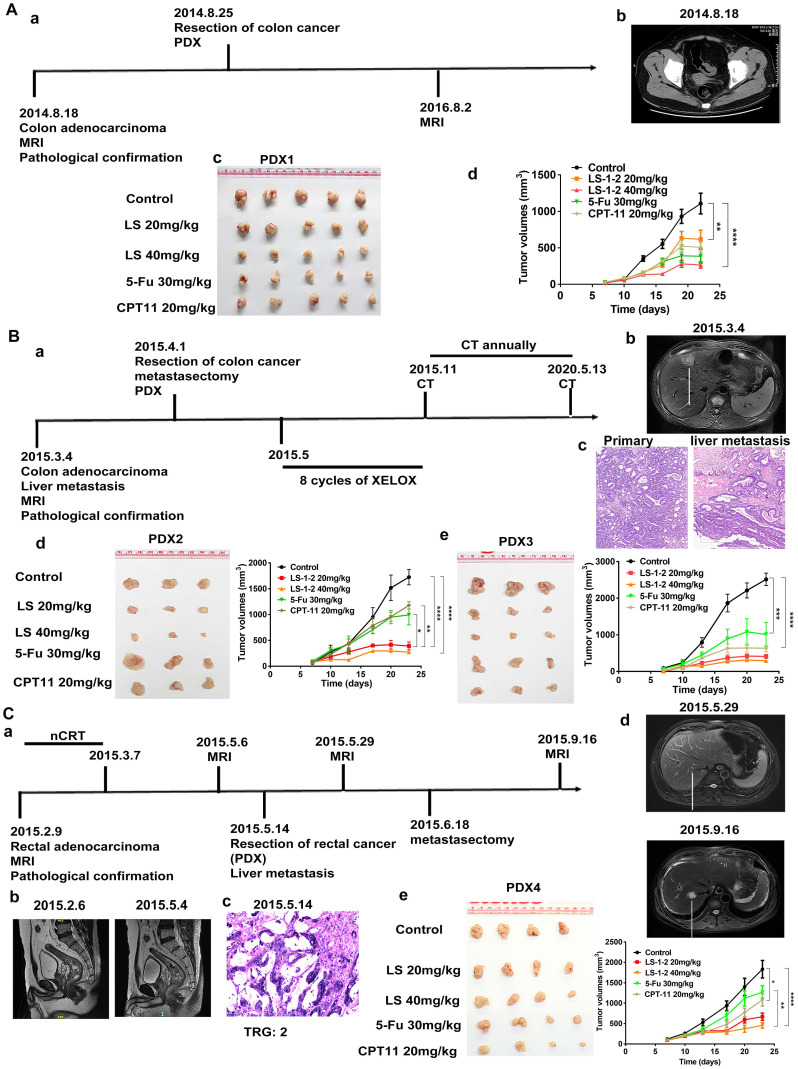
** LS-1-2 exhibits robust anti-tumor efficacy in CRC PDX models. A** a. The timeline of diagnosis and treatment procedures for patient Case1. b. MRI of pre-operation tumors from patient case 1. c. Representative image of PDX1 tumors in different groups. The mice in the treatment group were given PBS, LS-1-2 (20, 40 mg/kg, d1-d5/week), 5-FU (30 mg/kg, once weekly), or CPT-11 (20 mg/kg, once weekly) intraperitoneal injection for 18 days. d. The tumor volumes are plotted as the mean ± S.E.M. of n=6 mice per group. **B. a.** The timeline of diagnosis and treatment procedures for patient Case 2. **b**. MRI of pre-operation liver metastasis from patient case 2.** c.** The H&E staining of primary tumor and liver metastasis. Representative image of PDX2** (d)** from primary tumor and PDX3 **(e)** from liver metastasis in different groups. The tumor volumes are plotted as the mean ± S.E.M. of n=3 mice per group. **C. a.** The timeline of diagnosis and treatment procedures for patient Case 3. **b.** MRI of pre-and post-nCRT rectal primary tumors. **c.** The H&E staining of primary tumor and TRG. **d.** MRI of selected CT images of liver metastasis before and after metastasectomy. **e.** Representative image of PDX4 tumors in different groups. Tumor volumes and mouse weight were measured every three days. The tumor volumes are plotted as the mean ± S.E.M. of n=4 mice per group. The last time points were analyzed by using one-way ANOVA with a two-tailed unpaired *t*-test. *p < 0.05, **p < 0.01, ***p < 0.001, ****p < 0.0001.

PDX models exhibited considerable heterogeneity in drug response. PDX1, derived from a treatment-naïve primary colon cancer lesion, showed the strongest response to 5-FU, with 64.99% tumor inhibition (Figure 3A, Figure S4B). In contrast, PDX4, derived from a rectal cancer patient who received neoadjuvant chemoradiotherapy and subsequently developed liver and lung metastases, was the least sensitive to 5-FU, with only 31.93% tumor inhibition (Figure 3C, Figure S4H). PDX3, established from a metastatic lesion, exhibited the strongest response to CPT-11, with 81.15% tumor inhibition—approximately twice that of PDX2, which was derived from the primary lesion of the same patient (42.37% inhibition; Figure 3B, Figure S4F). PDX4 and PDX2 showed similar sensitivity to CPT-11 (Figure 3B, Figure S4D).

LS-1-2 (40 mg/kg) achieved higher tumor inhibition rates in primary colon cancer models (PDX1 and PDX2: 68.99% and 89.4%, respectively) than either 5-FU (64.99% and 57.20%) or CPT-11 (56.28% and 42.37%). In the liver metastasis-derived PDX3 model, LS-1-2 produced significantly greater tumor growth suppression (90.83% inhibition) compared to 5-FU (60.47%) and CPT-11 (81.15%). LS-1-2 also strongly inhibited tumor growth in the chemoradiation-resistant PDX4 model (81.51% inhibition), outperforming both 5-FU (31.93%) and CPT-11 (47.89%) (Figure 3, Figure S4).

### LS-1-2 Suppresses KRAS-Mutated CRC Liver Metastasis *In vitro* and *In vivo*

**Figure 4:**
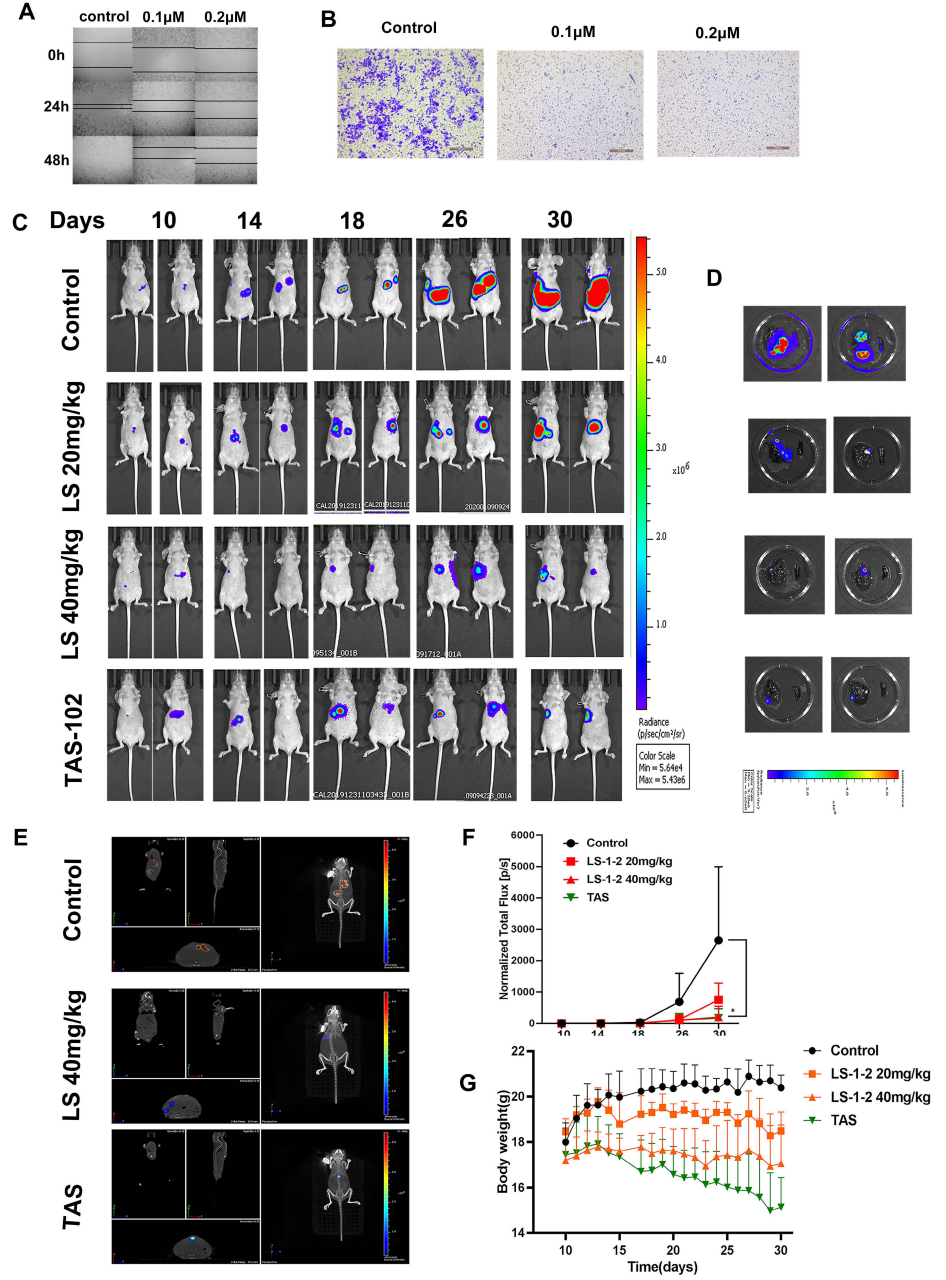
** LS-1-2 exhibits potent anti-tumor activity in a liver metastasis mouse model. A-B.** *In vitro* cell invasion and migration of HCT116 cells were detected by wound-healing and transwell assays. **C.** LS174T-Luciferase cells were implanted into the subcapsular area of the spleens of nude mice, and liver metastasis was monitored weekly by an *in vivo* imaging system. PBS or LS-1-2 was administered intraperitoneally. TAS-102 was delivered by gavage administration. Tumor growth was monitored weekly. **D**. Liver and spleen were imaged for tumor presence shortly after harvesting. **E**. Three-dimensional imaging of liver metastasis in mice. **F.** Quantification of bioluminescence. Data shown are presented as the mean ± S.D. The last time points were analyzed by using a two-tailed unpaired *t*-test. *p < 0.05. **G.** Body weight change in mice.

Based on the observed inhibition of metastatic KRAS-mutated CRC tumor growth following LS-1-2 treatment, we investigated whether LS-1-2 could directly prevent liver metastasis in KRAS-mutated colorectal cancer. LS-1-2 significantly suppressed HCT116 cell invasion and migration *in vitro* (Figure 4A-B). We further evaluated this effect* in vivo* using a mouse liver metastasis model. Intraperitoneal administration of LS-1-2 significantly reduced the dissemination of CRC liver metastases in a concentration-dependent manner (Figure 4C-E).

The efficacy of LS-1-2 (40 mg/kg) was comparable to that of TAS-102 (150 mg/kg, twice daily) in suppressing liver metastasis (Figure 4F). No body weight loss was observed in the LS-1-2 treatment group, whereas mice treated with TAS-102 exhibited a 10-14% reduction in body weight during the treatment period compared to baseline measurements (Figure 4G).

### LS-1-2 Reduces Colonosphere Formation and the Proportion of CSC

LS-1-2 markedly inhibited colonosphere formation in HCT116, HCT8, LS174T, SW480, and SW620 cell lines, as well as in two patient-derived cell lines (CASE1 and CASE3; Figure S5A-G). Furthermore, LS-1-2 significantly reduced the proportion of CD133+ cancer stem cells in HCT116/self cells (from 85.6% to 71.5%) and in patient-derived cell lines (PDC1: from 2.1% to 0.5%; PDC4: from 17.7% to 8.6%; Figure S5H).

In addition, LS-1-2 treatment resulted in a concentration-dependent downregulation of key stemness and drug resistance markers, including B-cell-specific Moloney leukemia virus insertion site 1 (BMI-1), Krüppel-like factor 4 (KLF4), multidrug resistance protein 1 (MDR1), NANOG, and ATP-binding cassette subfamily G member 2 (ABCG2; Figure S5I).

### LS-1-2 Regulates MAPK and PI3K/AKT-Induced FOXO Pathway

**Figure 5:**
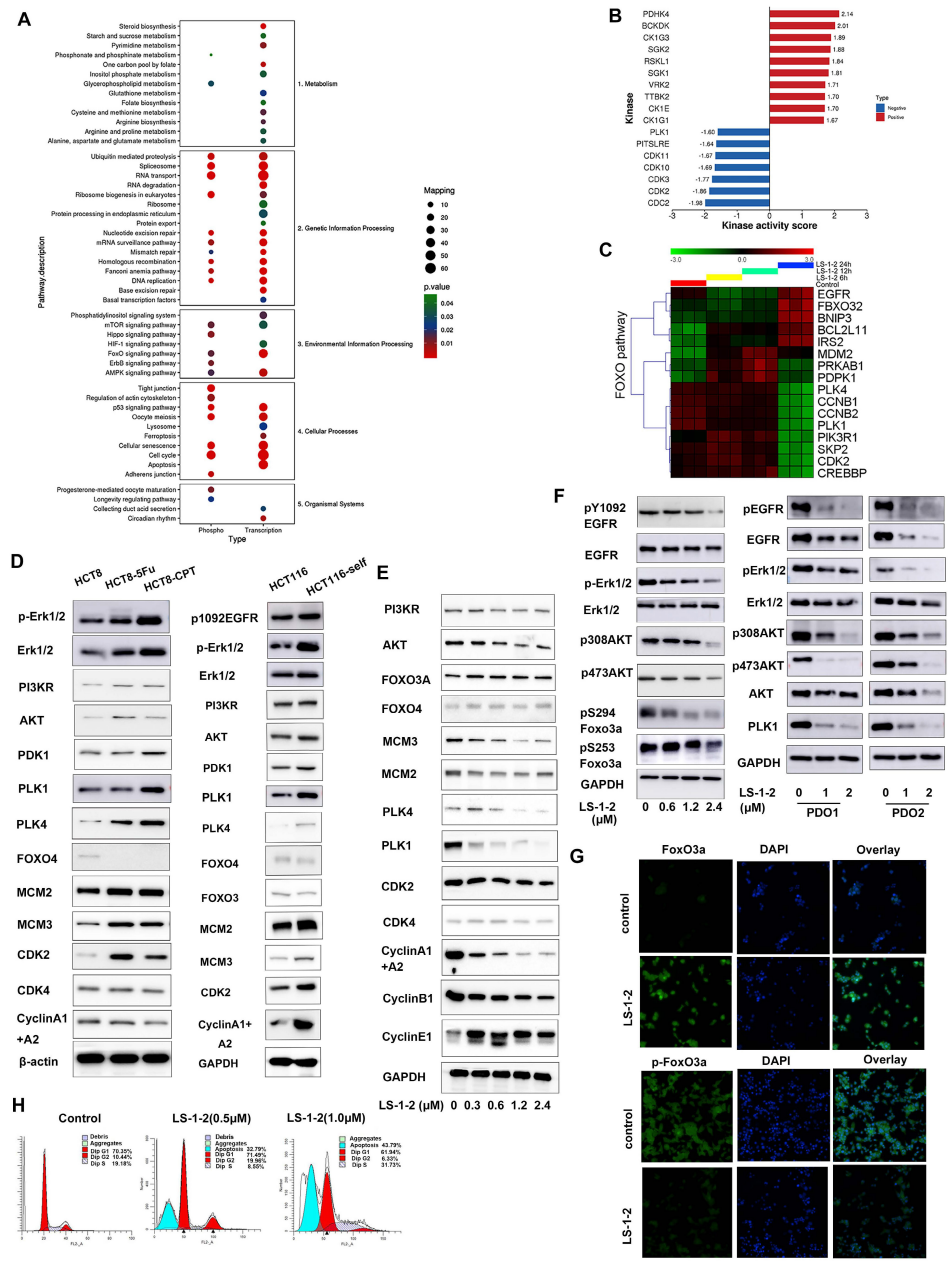
** LS-1-2 regulated MAPK and PI3K/AKT-induced FOXO pathway. A.** Enriched pathways for differentially phosphorylated proteins (phosphor) from quantitative phosphoproteomic and differentially expressed genes (transcription) from transcriptomics after LS-1-2 treatment using KEGG. **B.** Prediction of the effect of LS-1-2 on kinase activities. The GSEA method was used to predict kinase activities. **C.** Unsupervised clustering for 17 differentially expressed genes for FOXO pathway (p < 0.05) among control and LS-1-2 treatment at 0h, 12 h, and 24 h. **D.** The activity of MAPK and PI3K/AKT induced FOXO signaling pathway in chemo-resistant HCT8-5Fu, HCT8-CPT11, and HCT116/SELF cell lines. **E.** LS-1-2 treatment caused dose-dependent activation of FOXO proteins and inhibition of cell cycle regulation proteins. **F.** The effect of LS-1-2 on phosphorylation of EGFR, ERK, AKT, and FOXO3A in HCT116 cells and PDOs. **G.** FOXO3A and p-FOXO3A proteins in LS-1-2-treated or control HCT116 cells were detected using confocal microscopy. Green signals indicate Foxo3a or p-Foxo3a. Nuclei were counterstained with DAPI. Representative images of each sample are shown. **H.** The effect of LS-1-2 on the cell cycle was evaluated by flow cytometric analysis.

Integrated multi-omics analysis combining phosphoproteomics and RNA sequencing elucidated the mechanism of action of LS-1-2 in KRAS-mutant colorectal cancer. KEGG pathway enrichment analysis revealed that LS-1-2 affected several key pathways, including Cell cycle, p53 signaling, FOXO signaling, RNA transport, and metabolic pathways (Figure 5A). Phosphoproteomic profiling identified 2,253 differentially regulated phosphorylation sites (749 upregulated, 1,504 downregulated; Tables S6-7). Kinase activity prediction analysis indicated that LS-1-2 inhibited cell cycle kinases [cyclin-dependent kinase 1 (CDK1), cyclin-dependent kinase 2 (CDK2), polo-like kinase 1 (PLK1)] while activating p53-related pyruvate dehydrogenase kinase 4 (PDK4) (Figure 5B).

LS-1-2 upregulated FOXO pathway genes [F-box protein 32 (FBXO32), BCL2 interacting protein 3 (BNIP3), BCL2 like 11 (BCL2L11)] and downregulated cell cycle regulators [cyclin B1 (CCNB1), CDK2, PLK1] (Figure 5C). Western blot analysis confirmed that LS-1-2 restored FOXO3/4 expression in chemoresistant cell lines (HCT8-5Fu, HCT8-CPT11, and HCT116/self) while suppressing PLK1, PLK4, CDK2, cyclin A1/A2, and MAPK/PI3K pathway activation (Figure 5D-E). Additionally, LS-1-2 dose-dependently inhibited EGFR/ERK/AKT phosphorylation and promoted FOXO3A nuclear accumulation (Figure 5F-G).

Flow cytometry analysis demonstrated that LS-1-2 dose-dependently induced apoptosis, with apoptotic rates reaching 32.79% at 0.5 μM and 43.79% at 1.0 μM (Figure 5H). At 0.5 μM, LS-1-2 increased the proportion of cells in G2/M phase (19.96% vs. 10.44% in controls) while reducing the S-phase population (8.55% vs. 19.18%). In contrast, at the higher concentration of 1.0 μM, the compound markedly increased S-phase accumulation (31.73% vs. 19.18%) while decreasing the proportions of both G1-phase (61.9% vs. 70.35%) and G2/M-phase cells (6.33% vs. 10.44%) (Figure 5H). Transmission electron microscopy further revealed LS-1-2-induced ultrastructural alterations, including chromatin condensation and mitochondrial shrinkage at 12 hours, followed by nuclear chromatin lysis, mitochondrial swelling, and vacuolization at 24 hours, ultimately culminating in cell membrane dissolution (Figure S6).

These findings establish the dual mechanism of LS-1-2: it simultaneously targets EGFR and downstream signaling-driven chemoresistance pathways while reactivating FOXO-mediated tumor suppression.

### NMHC-IIA Is Identified as a Direct Target of LS-1-2

**Figure 6:**
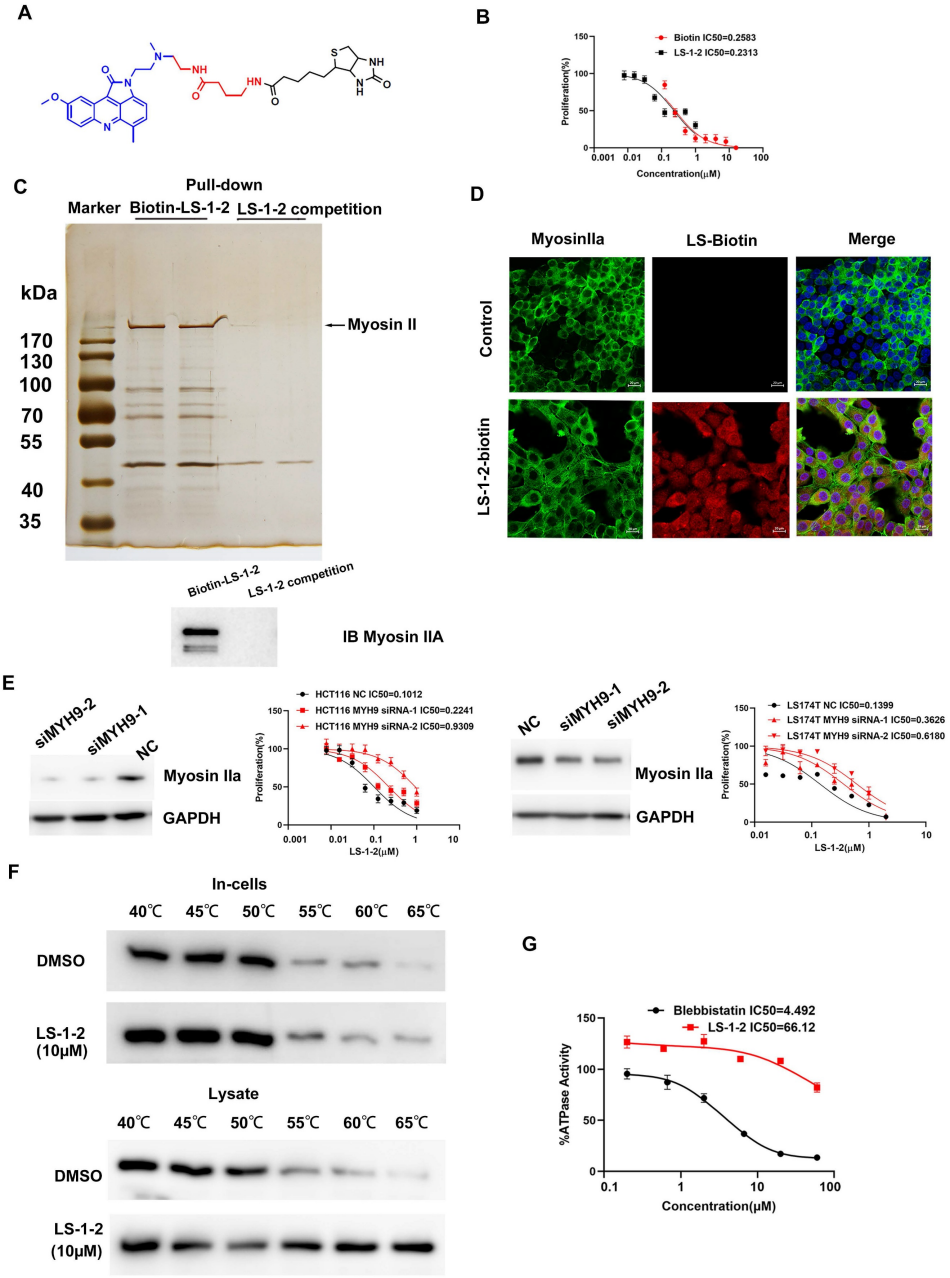
** NMHC IIA is identified as a direct target of LS-1-2. A.** The chemical structures of biotin-LS-1-2. **B.** Dose response of the proliferation of HCT116 cells exposed to LS-1-2 or LS-1-2-Biotin for 48 h. **C.** Identification of LS-1-2 target proteins using pull-down technology coupled with shotgun proteomics. **D.** HCT116 cells were treated with or without biotinylated-LS-1-2. Cells were stained with anti-NMHC IIa (labeling Myosin IIa, green) and anti-biotin (labeling LS-1-2-biotin, red, scale bars, 20 μm. n=3). **E.** The effect of MYH9 knockdown on the sensitivity of HCT116 and LS174T cells to LS-1-2. The effect of MYH9 knockdown on the expression of NMHC IIa. **F.** Cellular thermal shift assay (CETSA) using MYH9 overexpressed HCT116 intact cells or lysate were exposed to LS-1-2(10 μM). **G**. Titration curves of the *in vitro* ATPase assays for LS-1-2 and Blebbistatin inhibition of NMHC IIa.

To investigate the pharmacological target of LS-1-2, we synthesized chemical probes for affinity-based protein profiling (Figure 6A). A biotin-conjugated LS-1-2 probe maintained antitumor activity (Figure 6B) and selectively pulled down non-muscle myosin heavy chain IIA (NMHC-IIA, 226 kDa) through streptavidin affinity purification. This interaction was confirmed by SDS-PAGE with silver staining (Figure 6C), liquid chromatography-tandem mass spectrometry (LC-MS/MS; Table S8), and western blot analysis (Figure 6C).

Confocal microscopy demonstrated cytoplasmic colocalization of the LS-1-2-biotin probe with NMHC-IIA (Figure 6D). Knockdown of MYH9, the gene encoding NMHC-IIA, reduced cellular sensitivity to LS-1-2, supporting NMHC-IIA as a functional target (Figure 6E). Cellular thermal shift assay (CETSA) further validated direct binding, showing that LS-1-2 stabilized NMHC-IIA against thermal denaturation (Figure 6F). In contrast to the known inhibitor blebbistatin (IC₅₀ = 4.49 μM), LS-1-2 did not inhibit NMHC-IIA ATPase activity, indicating a mechanism of action independent of ATP-binding site interference (Figure 6G).

### NMHC IIA Phosphorylation at S1714 and S1943 Promotes CRC Cell Proliferation and Invasion

**Figure 7:**
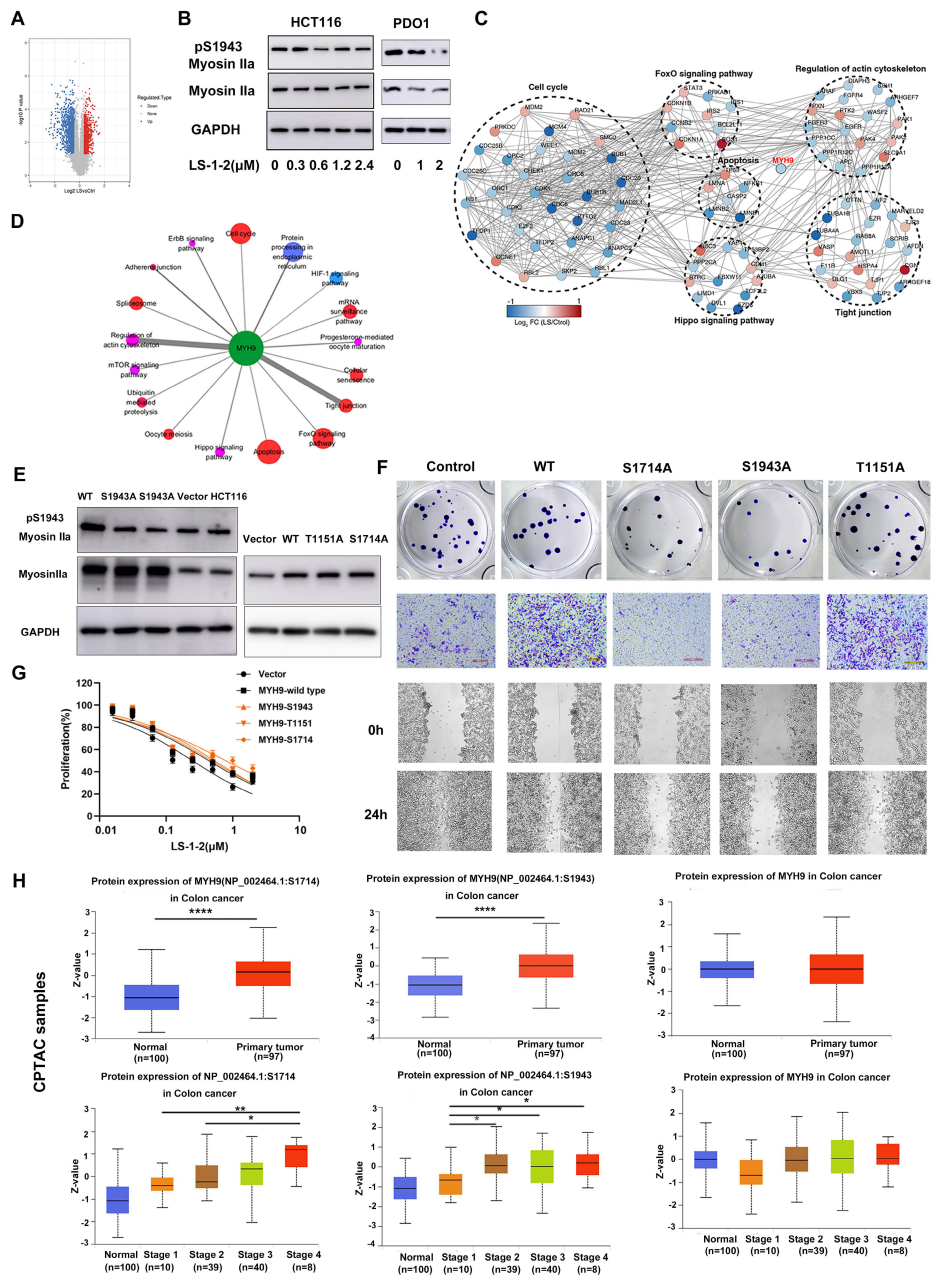
**NMHC IIA S1714 and S1943 phosphorylation promotes CRC cell proliferation and invasion and is reduced by LS-1-2 A.** A volcano plot shows that 2253 phosphorylation sites (found in a minimum of three biological replicates) change significantly (p-value < 0.05, FC > 1.3 or < 0.77) upon 2 μM LS-1-2 treatment for 18 h. **B.** The effect of LS-1-2 on the expression of myosin IIa and pS1943 myosin IIa in HCT116 cells and PDO1. **C.** Protein-protein interaction map of phosphoproteins whose sites are significantly changing upon LS-1-2 treatment. **D.** Pathways involved in NMHC IIA interacting proteins. **E.** The expressions of NMHC IIA and pS1943 NMHC IIA were detected in WT and NMHC IIA phosphorylation sites mutant HCT116 cells. **F.** The effects of NMHC IIA phosphorylation on CRC cell proliferation, invasion, and spreading were assayed by clonogenic, transwell, and wound healing assay. **G.** The drug sensitivity of the NMHC IIA phosphorylation site mutants and wild-type HCT116 cells to LS-1-2 was assayed by CCK8. **H.** The expression levels of NMHC IIA and phosphorylated NMHC IIA protein at S1714 and S1943 in tumor and non-tumor tissues. The expression levels of NMHC IIA and phosphorylated NMHC IIA protein at S1714 and S1943 in different TNM stages (data from CPTAC). The expression levels of phosphorylated NMHC IIA protein at S1714 and S1943 were higher in tumor tissues than in non-tumor tissues (p < 0.0001). The expression level of phosphorylated NMHC IIA protein at S1714 was higher in stage 4 than in stage 1-3 tumor tissues (p=0.003, p=0.053, and p=0.016, respectively). The expression level of phosphorylated NMHC IIA protein at S1943 was higher in stage 2-4 than in stage 1 tumor tissues (p=0.011, p=0.041, and p=0.032 respectively). *p < 0.05, **p < 0.01, ****p < 0.0001.

Analysis of global quantitative phosphoproteomic data revealed decreased phosphorylation of NMHC IIA at residues S1714, S1943, and T1151 in LS-1-2-treated HCT116 cells compared to controls (Figure 7A). Western blot analysis validated the reduction in NMHC IIA phosphorylation at S1943 in both HCT116 cells and PDO1 (Figure 7B; commercial antibodies were available only for pS1943 NMHC IIA). Phosphoproteomic profiling further indicated that NMHC IIA interacts with multiple signaling pathways enriched in proteins exhibiting LS-1-2-induced phosphorylation changes (Figure 7C).

To elucidate the signaling pathways affected by the LS-1-2-NMHC IIA interaction, we analyzed associations between altered phosphoproteins, differentially expressed mRNAs, and NMHC IIA using the STRING database, with pathway enrichment assessed via Kyoto Encyclopedia of Genes and Genomes (KEGG) annotation. This analysis identified perturbations in several key pathways, including Regulation of actin cytoskeleton, Tight junction, Cell cycle, Apoptosis, Hippo signaling, and FOXO signaling (Figure 7D).

Notably, CRC cell lines with lower basal S1943 phosphorylation demonstrated increased sensitivity to LS-1-2, as indicated by an inverse correlation between IC₅₀ values and phosphorylation levels (Figure S7 and Figure 2B). Mutational studies confirmed the functional importance of NMHC IIA phosphorylation, as HCT116 cells expressing S1714 phosphosite mutants exhibited reduced sensitivity to LS-1-2 (IC₅₀ = 0.7856 μM) compared to mock-transfected controls (IC₅₀ = 0.2487 μM; Figures 7F-G).

We further evaluated the clinical relevance of NMHC IIA phosphorylation in CRC using CPTAC data 14 Elevated phosphorylation at S1714 and S1943 was observed in CRC tumors compared to normal tissues and correlated with advanced TNM stage and metastatic progression. S1714 phosphorylation increased significantly in stage 4 tumors, while S1943 phosphorylation was elevated in stages 2-4. Total NMHC IIA protein levels remained unchanged across disease stages (Figure 7H).

### LS-1-2 Competitively Inhibits NMHC IIA Phosphorylation at S1943 and S1714 by CK2

**Figure 8:**
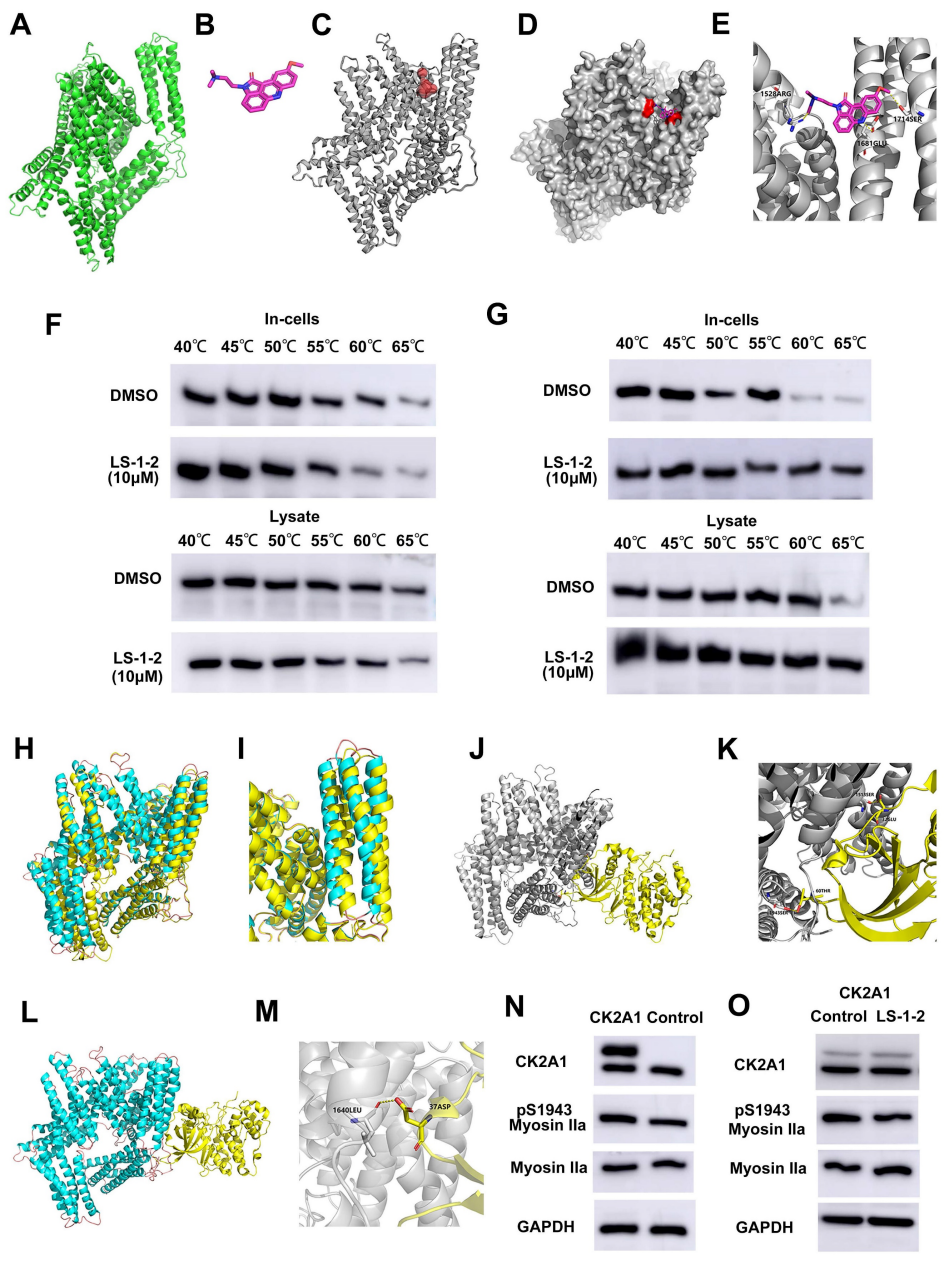
**Molecular dynamics.** Molecular docking of LS-1-2 on protein NMHC IIA. **A.** The 3D structure of the NMHC IIA was built using Rosetta8. **B.** Chemical structure of LS-1-2. **C.** The active sites NMHC IIA were predicted using CASTp software. **D.** The LS1-2 ligand docked conformation with NMHC IIA. **E.** The interaction residues between the LS1-2 and NMHC IIA. **F**. CETSA using MYH9-S1714A overexpressed HCT116 intact cells or lysate were exposed to LS-1-2(10 μM). **G**. CETSA using MYH9-S1943A overexpressed HCT116 intact cells or lysate were exposed to LS-1-2(10 μM). **H.** The conformational change between the initial NMHC IIA structure (yellow) and the final structure (blue) after the NMHC IIA docked with LS-1-2 was compared to detect the structure alteration. **I.** The conformational change of the alpha helix regions belonging to the CK2A1 binding domain. **J.** The initial NMHC IIA structure binds to CK2A1 before combining with LS-1-2. **K.** The details of the binding area in the J. **L**. The final NMHC IIA structure binds with CK2A1 after combining with LS-1-2. **M.** The details of the binding area in the L. **N.** The expression levels of CK2A1, pS1943 Myosin IIa, and Myosin IIa in CK2A1-overexpressing HCT116 cells and control cells. **O**. The effect of LS-1-2(2μM) on the expressions of CK2A1, pS1943 Myosin IIa, and Myosin IIa in CK2A1-overexpressing HCT116 cells.

Using homology modeling and CASTp analysis, we predicted the active sites of NMHC IIA (ligand-binding domain volume: 71.031 Å) and confirmed through molecular docking that LS-1-2 binds to these regions (Figures 8A-E). CETSA demonstrated that LS-1-2 stabilizes NMHC IIA through direct interaction with S1714, as mutation of this residue abolished thermal stabilization (Figure 8F), whereas S1943 mutation did not affect LS-1-2-induced stabilization (Figure 8G). Molecular dynamics simulations revealed that LS-1-2 binding at S1714 creates steric hindrance that prevents phosphorylation at this site (Figure 8E). Given previous reports that NMHC IIA S1943 is phosphorylated by casein kinase II (CK2) 15. LS-1-2 binding induced conformational changes in the CK2A1-binding alpha helices of NMHC IIA (Figures 8H-I), reducing the CK2A1-NMHC IIA binding energy from -92.08 kJ/mol to -48.73 kJ/mol (Figures 8J-M). Overexpression of CK2A1 increased S1943 phosphorylation, and this effect was inhibited by LS-1-2 treatment (Figures 8N-O). These results indicate that LS-1-2 disrupts CK2A1-mediated phosphorylation at S1943 through allosteric interference with the kinase-substrate interaction.

### NMHC IIA Phosphorylation at S1714 and S1943 Promotes YAP-Mediated Activation of FOXO/PLK1 Signaling and Is Suppressed by LS-1-2

**Figure 9:**
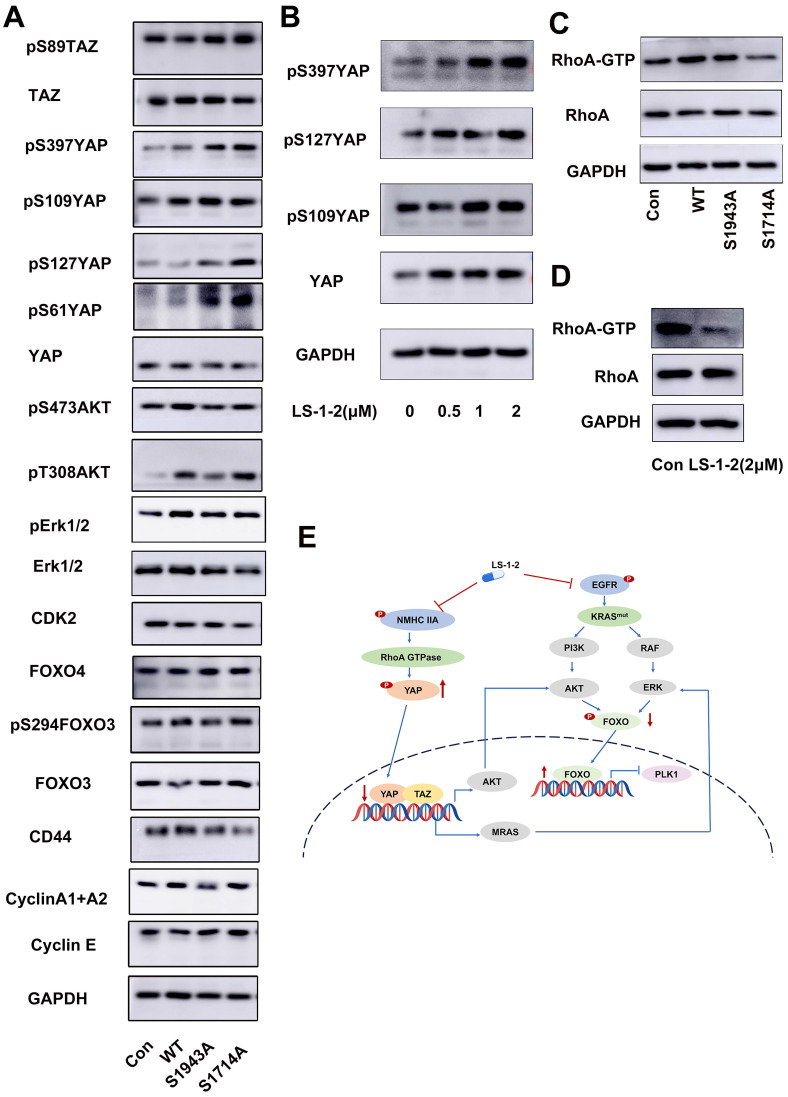
**NMHC IIA S1714 and S1943 phosphorylation-mediated YAP activity induces activation of ERK and AKT and is suppressed by LS-1-2. A.** The effect of NMHC IIA phosphorylation on Hippo signaling and PI3K/AKT, ERK pathway. **B.** The effect of LS-1-2 on the Hippo signaling pathway.** C**. The effect of NMHC IIA phosphorylation on the active RhoA was analyzed using a RhoA-GTP pull-down assay. **D.** The effect of LS-1-2 on the active RhoA was analyzed by RhoA-GTP pull-down assay. **E.** Model depicting the mechanism by which LS-1-2 inhibits PI3K/AKT and ERK signaling pathways, thereby suppressing PLK1 expression.

We investigated the effect of NMHC IIA phosphorylation at S1714 and S1943 on MAPK, AKT, and FOXO signaling pathways. Overexpression of wild-type MYH9 increased phosphorylation of AKT, ERK1/2, and FOXO3 compared to control. In contrast, phosphodeficient mutations (S1943A and S1714A) failed to upregulate phosphorylation of these proteins. Both S1943A and S1714A mutations also suppressed cyclin A1/A2 expression while upregulating cyclin E levels relative to wild-type MYH9 (Figure 9A).

We further examined the relationship between NMHC IIA phosphorylation and Hippo pathway regulation. The S1943A and S1714A mutations promoted phosphorylation of Yes1-associated transcriptional regulator (YAP) and transcriptional coactivator with PDZ-binding motif (TAZ), resulting in decreased YAP protein levels. LS-1-2 treatment similarly enhanced YAP phosphorylation (Figure 9B).

Rho GTPase pull-down assays demonstrated that wild-type NMHC IIA significantly enhanced RhoA activity in HCT116 cells, whereas both S1943A and S1714A mutations inhibited Rho activation. LS-1-2 treatment also substantially suppressed Rho activity in cellular assays (Figure 9C-D).

### NMHC IIA Phosphorylation Is Inversely Correlated with Prognosis and Adjuvant Chemotherapy Outcomes in CRC

We further evaluated the association between NMHC IIA phosphorylation at S1943 and clinical prognosis. Immunohistochemical analysis revealed that pS1943-myosin was localized to both the cell membrane and cytoplasm of tumor cells (Figure S8A). Examination of 12 paired samples from patients with and without KRAS mutations showed elevated p-NMHC IIA levels in KRAS-mutant specimens (Figure S8B). The extent of NMHC IIA phosphorylation positively correlated with advanced TNM stage, liver metastasis (M stage), poor differentiation, and CD44 expression (Table S9). Elevated NMHC IIA phosphorylation in tumor tissues was significantly associated with reduced overall survival (p = 0.049, log-rank test; Figure S8C). Furthermore, increased NMHC IIA phosphorylation correlated with shorter disease-free survival in stage I-III CRC patients (P = 0.04; Figure S8D).

## Discussion

To date, with the exception of recent successes in targeting the relatively uncommon KRAS-G12C mutation in CRC, therapeutic targeting of KRAS in CRC and other malignancies has seen limited progress 16. Our RNA sequencing analysis of PDOs demonstrated that KRAS mutations drive global transcriptional activation across the genome, a finding validated at single-cell resolution. This observation is consistent with previous reports 17. Hypertranscription has been associated with poorer prognosis in various tumor types, including CRC 18.

Patient-derived cancer models such as organoids and PDXs are increasingly recognized as valuable tools for drug screening and cancer therapeutic development 19, 20. In this study, we utilized multiple PDOs derived from KRAS-mutated refractory metastatic CRC as drug screening platforms. Screening of an FDA-approved anticancer drug library identified the acridine derivative amsacrine as a potent inhibitor of both KRAS-mutant PDOs and cell lines. Acridone derivatives are known to interact with multiple targets, including topoisomerases, telomerase, and CDKs. These interactions disrupt key oncogenic processes such as DNA replication, transcription, proliferative signaling, and genomic stability, ultimately inducing apoptotic or cytotoxic responses in malignant cells 21.

We synthesized and screened a series of novel acridine derivatives to address limitations in solid tumor penetration and tolerability. This effort identified LS-1-2, an acridine derivative demonstrating potent anti-tumor and anti-metastatic activity against KRAS-mutated CRC in both *in vitro* and *in vivo* models. Multi-omics analysis revealed that LS-1-2 significantly modulates the FOXO signaling pathway. Forkhead box O (FOXO) transcription factors play critical roles in regulating proliferation, apoptosis, differentiation, and cell cycle progression 22. Further investigation demonstrated that LS-1-2 inhibits EGFR and downstream MAPK/ERK and PI3K/AKT signaling pathways, which are known negative regulators of FOXO transcription factors.

LS-1-2 was found to suppress the expression and kinase activity of key cell cycle regulators including PLK1, CDK2, and CDK4. KRAS mutations drive elevated global transcription, increasing cellular dependence on essential cell cycle proteins such as PLK1, anaphase-promoting complexes, COP9 signalosomes, and proteasomal components. This dependency creates opportunities for synthetic lethality by targeting these critical cell cycle regulators in KRAS-mutant cells 23. Preclinical and clinical evidence supports PLK1 inhibition as a promising therapeutic strategy for KRAS-mutated colorectal cancer 24.

Mechanistic studies revealed that LS-1-2 binds directly to NMHC IIA and inhibits its phosphorylation, consequently suppressing Hippo pathway activation. NMHC II regulates cell motility through dynamic interactions with F-actin and microtubules 25, and its aberrant activation promotes tumor progression, establishing NMHC II as a therapeutic target in invasive cancers 26-30. Myosin II activation has been implicated in resistance to MAPK pathway inhibitors, and such resistance can be overcome by inhibiting myosin II activity 31.

NMHC-IIA phosphorylation plays a critical role in myosin assembly and disassembly, facilitating myosin-IIA recruitment to the lamella during cell spreading 32, 33. In this study, we observed elevated phosphorylation of NMHC-IIA at S1714 and S1943 in colorectal cancer tissues, which correlated with tumor progression and distant metastasis. Phosphorylation at S1943 was notably higher in KRAS-mutant CRC tissues compared to KRAS wild-type specimens. Functional assays confirmed that NMHC-IIA phosphorylation at S1943 and S1714 promotes CRC cell proliferation, invasion, and migration.

Further investigation revealed that NMHC-IIA phosphorylation activates the YAP signaling pathway and modulates ERK, AKT, and FOXO signaling. LS-1-2 binding to NMHC-IIA induces conformational changes in the protein, resulting in impaired phosphorylation at S1943 and S1714 by CK2A1.

Previous studies have established that the PI3K/AKT and MAPK/ERK pathways can be modulated by Hippo signaling activation. YAP enhances AKT phosphorylation 34 and directly upregulates Pik3cb expression through TEAD transcription factors, thereby activating the PI3K-AKT pathway 35. Additionally, YAP1 has been shown to induce ERK reactivation via the RAS-related GTPase MRAS following KRAS G12C inhibitor treatment 36. The observed reduction in ERK and AKT expression and activity upon LS-1-2 treatment may therefore be mediated through inhibition of YAP signaling.

Trifluridine/tipiracil (TAS-102) received US FDA approval in 2015 for the treatment of refractory mCRC. As a single-agent regimen for patients with refractory mCRC, TAS-102 has demonstrated a moderate improvement in overall survival 37, 38. In our comparative evaluation of anti-metastatic efficacy, LS-1-2 showed activity comparable to TAS-102. Notably, LS-1-2 treatment did not induce body weight loss, whereas significant weight reduction was observed in the TAS-102 group.

Our findings also reveal an additional biological dimension to NMHC IIA function. Non-muscle myosin IIA has been implicated in protein trafficking to biological membranes, where it participates in diverse processes including exocytosis regulation and organelle localization 39. Previous studies have shown that NMIIA interacts with RIPK1 to facilitate transport of the RIPK1-ceramide complex to the plasma membrane, leading to formation of large membrane pores termed ceramidosomes that mediate membrane blebbing and necroptosis 40. In line with these observations, we found that LS-1-2 induces a methuosis-like cell death phenotype, suggesting that its binding to NMHC IIA disrupts lipid bilayer integrity, promoting large vacuole formation and necroptotic cell death.

In conclusions, this study demonstrates that KRAS-mutated colorectal cancer cells develop a hypertranscription phenotype that contributes to aggressive tumor progression. Through systematic pharmacological screening, we identified a novel acridine derivative, LS-1-2, that effectively suppresses KRAS-driven tumor growth and metastasis. Mechanistically, LS-1-2 counteracts oncogenic hypertranscription by inhibiting NMHC IIA phosphorylation, thereby reactivating the FOXO tumor suppressor pathway and impairing transcriptional addiction. These findings establish LS-1-2 as a promising preclinical candidate for targeting transcriptionally addicted KRAS-mutant malignancies and provide a rationale for therapeutic targeting of the NMHC IIA/FOXO axis in hypertranscription-associated cancers.

## Supplementary Material

Supplementary materials and methods, figures and tables 1-4, 9-10.

Supplementary tables 5-8.
