# Supplementary material for: KRAS-Driven Hypertranscription and Metastatic Dissemination in Colorectal Cancer Could be Overcome by Targeting the NMHC IIA/ PLK1 Signaling Axis with a Novel Acridine Derivative
Source: Int J Biol Sci. 2026 Jan 1;22(1):500–18. doi: 10.7150/ijbs.121816 (PMC12681934; doi:10.7150/ijbs.121816)
Supplement: Supplementary file 1 — Supplementary materials and methods, figures and tables 1-4, 9-10. [file ijbsv22p0500s1.pdf]

Supplementary Materials for

KRAS-Driven Hypertranscription and Metastatic Dissemination in Colorectal Cancer Could be Oovercome by Targeting the NMHC IIA/ PLK1 Signaling Axis with a Novel Acridine Derivative

Dengbo Ji[a,1], Haizhao Yi[f,1], Ming Li[a,1], Zhaoya Gao[b,1], Rui Yang[c,1], Jingying Jia[a,1], Xinxin Cui[a], Can Song[d,e], Hanyang Wang[a], Mengyuan Shi[a], Yuhao Yan[c], Tongtong Geng[c], Xiangbao Meng[c*], Zhongjun Li[c], Jin Gu[a, b, d, e*]

Correspondence to: zlguj@bjmu.edu.cn, xbmeng@bjmu.edu.cn

This PDF file includes:

Materials and Methods
Tables S1-4, 9-10
Figures S1-12

Material and methods

Patients and Samples

CRC tissue samples used in this study were obtained from Peking University Shougang Hospital. The Ethics Review Committees of Peking University Shougang Hospital gave their approval for the collection and use of the samples in compliance with the Declaration of Helsinki (IRBK-2021-026-01, 2021.7). All patients were informed before the study began, and each participant signed a consent form. Cohort 1 consisted of 294 patients who had surgical resections; all of the tissues from these patients included CRC and the surrounding non-tumor tissues. These specimens underwent microdissection and paraffin embedding to produce tissue microarrays (TMAs) for immunohistochemistry. Every sample was taken before undergoing any preoperative radiation or chemotherapy, and it was confirmed that at least 80% of the tumor cells were present. The study did not include cases of familial adenomatous polyposis colorectal cancer. Follow-up data is available for every sample. Table S1 provides an overview of these patients' clinical features.

For PDXs, a few primary CRC tissues with KRAS mutations were chopped up and separated into individual cells using the Human Tumor Dissociation Kit (Miltenyi Biotech, 130-095-929) before being xenografted into NOD SCID mice. For PDOs, several fresh surgically removed KRAS-mutant or wild type colorectal cancer tissues were employed.

Table S2 and Figure S1 provide an overview of the PDXs' clinical features. Table S3 provides an overview of the PDOs' clinical features.

Synthesis of chemicals

2-(2-(dimethylamino)ethyl)-9-methoxy-5-methylpyrrolo[2,3,4-kl]acridin-1(2H)-one (LS-1-2)

As shown in Figure S9A, a solution of 4-methylcyclohexane-1,3-dione (146 mg, 1.3 mmol) and N^1, N^1-dimethyl ethane -1,2-diamine (143 μL, 1.3 mmol) in toluene (5 mL), was added L-proline (12 mg, 0.1 mmol). The mixture was heated to reflux for 4 hours before adding 5-methoxyindoline-2,3-dione (177 mg, 1.0 mmol). The reaction was carried out for an additional 6 hours, then evaporated to dryness. The residue was subjected to flash chromatography (CH_2Cl_2-CH_3OH=30:1) to furnish LS-1-2 as an orange solid (168 mg, 50.0%). ^{1}H NMR (400 MHz, $CDCl_3$) δ 8.32 (d, J = 9.5 Hz, 1H), 8.08 (d, *J* = 2.8 Hz, 1H), 7.53 (dd, *J* = 9.5, 2.8 Hz, 1H), 7.39 (dd, *J* = 6.9, 1.0 Hz, 1H), 6.85 (d, *J* = 6.9 Hz, 1H), 4.10 (t, *J* = 7.1 Hz, 2H), 4.05 (s, 3H), 2.85 (s, 3H), 2.74 (t, J = 7.1 Hz, 2H), 2.38 (s, 6H) (Figure S10). ^{13}C NMR (101 MHz, $CDCl_3$) δ 168.39, 160.20, 148.44, 144.05, 137.42, 132.51, 132.08, 131.17, 129.49, 125.05, 124.54, 119.60, 105.33, 99.85, 57.58, 55.95, 45.69, 38.65, 16.17 (Figure S11). HR-ESI-MS: Cald for $C_{20}H_{22}N_3O_2$, 336.17065 $[M+H]^+$, found: 336.17096 $[M+H]^+$ (Figure S12) [1].

Synthesis of biotin-conjugated LS-1-2 (biotin-LS-1-2)

Step 1

As shown in Figure S9B, a solution of 4-methylcyclohexane-1,3-dione (146 mg, 1.3 mmol) and *tert*-butyl (2-((2-aminoethyl)(methyl)amino)ethyl)carbamate (282 mg, 1.3 mmol) in toluene (5 mL), was added L-proline (12 mg, 0.1 mmol). The mixture was heated to reflux for 4 hours before adding 5-methoxyindoline-2,3-dione (177 mg, 1.0 mmol). The reaction was carried out for an additional 6 hours, then evaporated to dryness. The residue was subjected to flash chromatography (CH_2Cl_2-CH_3OH=30:1) to furnish

intermediate **1** an orange solid (181 mg, 39.0%).

Step 2

To an ice-cold solution of intermediate 1 (146 mg, 0.39 mmol) in CH_2Cl_2 (3 mL) was added TFA (0.5 mL). The mixture was warmed to room temperature and stirred for 5 hours, then concentrated under reduced pressure. The residue was dissolved in CH_2Cl_2 (5 mL) and washed with a saturated $NaHCO_3$ solution. The organic layer was dried over Na_2SO_4, concentrated, and purified by flash chromatography (CH_2Cl_2-CH_3OH = 15:1) to produce intermediate **2** (110 mg, 77%) as an orange solid.

Step 3

To a solution of D-biotin (600 mg, 2.46 mmol) in DMF, was added EDC (560 mg, 0.29 mmol) and *N*-hydroxy succinimide (340 mg, 0.29 mmol). The mixture was stirred at room temperature overnight and then concentrated under reduced pressure. The residue was filtered and washed with EtOH-HOAc-H_2O (95:1:4). The obtained intermediate **3** (775 mg, 92%) was used for the next reaction without further purification.

Step 4

A solution of intermediate **2** (110 mg, 0.30 mmol) and **3** (123 mg, 0.36 mmol) in dioxane (5 mL) was stirred at room temperature for 24 hours. The mixture was concentrated under reduced pressure and re-dissolved in CH_2Cl_2 (10 mL). The organic layer was washed with saturated $NaHCO_3$ solution, dried over Na_2SO_4, concentrated, and purified by flash chromatography (CH_2Cl_2-CH_3OH = 15:1) to produce biotin-LS-1-2 (130 mg, 73.4%) as an orange solid. ^{1}H NMR (400 MHz, $CDCl_3$) δ ^{1}H NMR (400 MHz, Chloroform-*d*) δ 8.34 (d, J = 9.6 Hz, 1H), 8.02 (d, J = 2.9 Hz, 1H), 7.54 (dd, J = 9.6, 2.9 Hz, 1H), 7.40 (d, J = 7.0 Hz, 1H), 6.87 (d, J = 7.0 Hz, 1H), 6.45 (s, 1H), 6.26 (s, 1H), 5.52 (s, 1H), 4.50 (t,

J = 6.4 Hz, 1H), 4.26 (t, J = 6.3 Hz, 1H), 4.14 – 4.04 (m, 4H), 3.62 (q, J = 7.4 Hz, 1H), 3.28 (s, 2H), 3.05 – 2.98 (m, 1H), 2.93 – 2.85 (m, 2H), 2.84 (s, 3H), 2.75 (d, J = 12.8 Hz, 1H), 2.64 (s, 2H), 2.38 (s, 3H), 2.30 (s, 2H), 2.19 (d, J = 1.9 Hz, 1H), 1.77 (brs, 2H), 1.55-1.42 (m, 2H), 1.40 – 1.12 (m, 4H). ^{13}CNMR (101 MHz, $CDCl_3$) δ 173.05, 168.65, 163.56, 160.23, 148.38, 143.92, 137.23, 132.53, 131.51, 129.63, 125.49, 125.25, 124.56, 119.59, 105.60, 99.76, 61.65, 60.11, 55.98, 55.81, 55.43, 52.85, 42.20, 40.60, 38.37, 36.40, 35.39, 30.97, 27.97, 25.39, 16.28. TOF-MS: Cald for $C_{31}H_{39}N_6O_4S$, 591.2753 $[M+H]^+$, found: 591.2551[2].

Patient-derived organoid culture

Briefly, fresh, surgically resected CRC samples were washed in cold PBS with penicillin/streptomycin (GIBCO, 15140-122) and then minced into tiny pieces in a sterile dish on ice. The tissue fragments were then subjected to enzymatic digestion in a 10 mL Gentle Cell Dissociation Reagent (Stemcell, 07174) on a rocking platform at 4 ℃ for 30 min. Tumor cells were collected after centrifugation and filtered by a 70 µm strainer. The cells that were not filtered out were resuspended in Intesticult Organoid Growth Medium (Stemcell,06010) and cultured at 37 °C in a humidified atmosphere of 5% CO_2.

Patient-derived cell line (PDC) establishment and culture

PDX tissues were dissociated into single-cell suspension by mechanical dissociation using the Gentle MACS Dissociator (Miltenyi Biotec), and the extracellular matrix was enzymatically degraded using the Human Tumor Dissociation Kit (Miltenyi Biotec). The cell suspension was centrifuged three times and suspended in DMEM/F12 medium containing 10% FBS. Finally, the cell suspension was filtered through a 70-micron cell filter (Meltyi-BioTeC). The cells that were not filtered out were resuspended in

DMEM/F12 medium containing B27, bFGF, and EGF, and were then cultured at 37 °C in a humidified atmosphere of 5% CO_2. Growing cell lines were further subcultured and three consecutive freeze-thaw cycles were carried out to ensure the stability of the cell lines. The growing cell lines were stocked at low passages by cryopreservation.

Cell transfection

The Lipofectamine 2000 (Invitrogen, USA) was used to transfect cells with plasmids or siRNAs. The MYH9-targeting small interfering RNAs (siRNAs) were synthesized by RiboBio Co. Ltd. The siRNA sequences are shown in Table S8. The MYH9 plasmids with or without mutations on phosphorylation sites (S1714, S1943, and T1151) were constructed by GeneChem (Shanghai, China). The complementary DNA (cDNA) for WT MYH9 and MYH9 mutants in phosphorylation sites S1714, S1943, and T1151 were amplified by a polymerase chain reaction and ligated into GV657 plasmid. The special single base pairs of the triplet encoding S1714, S1943, and T1151 within the MYH9 gene were mutated, resulting in substitution to alanine (A, which blocks phosphorylation) in the derived protein. The CSNK2A1 plasmids were constructed by GeneChem (Shanghai, China).

Cell cytotoxicity Assay

The cells were treated with serial dilutions of the drug in triplicate after being plated in 96-well plates with 10,000 cells per well. A cell counting kit-8 (CCK-8) (Dojido, Kumamoto, Japan) test was used to evaluate cell viability 48 hours following treatment with LS-1-2, 5-FU, or CPT-11. Untreated controls were used to standardize the proportion of cell survival in treated samples. With GraphPad Prism 5.0 (San Diego, CA), the IC50 was determined.

In vitro cell growth, spreading, motility, and invasion assays

CCK-8 (Dojindo, Tokyo, Japan) was used to measure cell growth following the manufacturer's instructions. For the clonogenic assay, cells were low-density plated in 6-well plates. After being fixed, cells were stained using 1% crystal violet solution (Sigma-Aldrich). An assay for wound healing was used to assess cell spreading. After scratching, time-lapsed microphotos were taken at 0 and 24 hours to evaluate wound closure. With or without Matrigel, a Boyden chamber test was used to assess cell invasion or motility. Three randomly chosen fields of the fixed cells were photographed, and the number of cells was determined. Each experiment was conducted three times independently.

Spheroid formation assay

Spheroid formation was performed by plating 100 cells per well into a 96-well ultra-low attachment plate (Corning Incorporated Life Sciences, Acton, MA) in serum-free DMEM/F12 medium (Invitrogen), supplemented with B27 (1:50; Gibico), 50 ng/mL bFGF, 50 ng/mL EGF (Invitrogen), and 1% methylcellulose (Sigma-Aldrich) named as complete culture. Cells were incubated in a CO_2 incubator for 2–3 weeks and spheres were counted under a stereomicroscope (Olympus, Shinjuku-ku, Tokyo, Japan). For the drug assay, the culture medium was replaced with a complete culture medium containing a series of concentrations of LS-1-2.

PI labeling and FACS

The CRC cells were seeded into 60-mm culture dishes at 1×10^5 cells/mL and cultured until attached. The cells were serum-starved for 12 h with the serum-free medium 1640, followed by fresh media change containing 10% FBS with or without LS-1-2 (0.5 and 1.0

μM) and incubated for 24 h. Cell cycle distribution was evaluated by PI staining of nuclei and flow cytometric analysis. The cells were harvested, washed with ice-cold PBS, and resuspended with PI. The final mixture of PI stain was then applied to a FACSAria II flow cytometer (BD Biosciences, San Jose, CA) for analysis (approximately 10,000 events).

RNA isolation and microarray analysis

Total RNA from six cell samples was extracted using TRIZOL Reagent (Cat#15596-018， Life Technologies, Carlsbad, CA, US) following the manufacturer's instructions and checked for a RIN number to inspect RNA integrity by an Agilent Bioanalyzer 2100 (Agilent Technologies, Santa Clara, CA, US). Qualified total RNA was further purified by RNeasy micro kit (Cat#74004, QIAGEN, GmBH, Germany) and RNase-Free DNase Set (Cat#79254, QIAGEN, GmBH, Germany). Total RNA was amplified, labeled, and purified by Affymetrix WT PLUS Reagent Kit (Cat#902280, Affymetrix, Santa Clara, CA, US) according to the manufacturer's instructions to obtain biotin-labeled cDNA. The labeled cDNA targets were then hybridized on slides of Affymetrix GeneChip® Human Transcriptome Array 2.0. After hybridization, slides were scanned Affymetrix GeneChip® Scanner 3000 (Cat#00-00213, Affymetrix, Santa Clara, CA, US). Data were extracted using Command Console Software 4.0 (Affymetrix, Santa Clara, CA, US). Then, raw data were normalized by Expression Console. The microarray experiments were performed following the protocol of Affymetrix Inc. (Shanghai Biotechnology Corporation).

Quantitative phosphoproteomic analysis

The collected samples were added with 4 times the volume of 10% TCA/acetone and

kept at -20 °C for 4 h. After centrifuging at 4 °C for 5 min, the supernatant was discarded, and the precipitate was washed with precooled acetone three times, air dried, and then finally redissolved with 8 M urea. The protein concentration was determined using the BCA kit according to the manufacturer's instructions, and the protein sample was then diluted by adding 100 mM TEAB to urea concentration of less than 2 M. Finally, trypsin was added at a 1:50 trypsin-to-protein mass ratio for the first digestion overnight and a 1:100 trypsin-to-protein mass ratio for a second 4 h-digestion. The protein solution was then reduced with 5 mM dithiothreitol for 30 min at 56 °C and alkylated with 11 mM iodoacetamide for 15 min at room temperature in darkness. After trypsin digestion, peptides were desalted by Strata X C18 SPE column (Phenomenex) and vacuum-dried. Peptides were reconstituted in 0.5 M TEAB and processed according to the manufacturer's protocol for the TMT kit/iTRAQ kit. Briefly, one unit of TMT/iTRAQ reagent was thawed and reconstituted in acetonitrile. The peptide mixtures were then incubated for 2 h at room temperature and then pooled, desalted, and dried by vacuum centrifugation. The tryptic peptides were fractionated into fractions by high pH reverse-phase HPLC using Agilent 300Extend C18 column (5 μm particles, 4.6 mm ID, 250 mm length). Briefly, peptides were first separated with a gradient of 8% to 32% acetonitrile (pH 9.0) over 60 min into 60 fractions. Then, the peptides were combined into 6 fractions and dried by vacuum centrifuging. The tryptic peptides were dissolved in 0.1% formic acid (solvent A) and directly loaded onto a homemade reversed-phase analytical column (15-cm length, 75 μm i.d.). The gradient was comprised of an increase from 6% to 23% solvent B (0.1% formic acid in 98% acetonitrile) over 26 min, 23% to 35% over 8 min, and climbing to 80% in 3 min then holding at 80% for the last 3 min, all

at a constant flow rate of 450 nL/min on an EASY-nLC 1200 UPLC system. The peptides were subjected to NSI source followed by tandem mass spectrometry (MS/MS) in Q ExactiveTM Plus (Thermo) coupled online to the UPLC. The electrospray voltage applied was 2.0 kV. The m/z scan range was 350 to 1800 for a full scan, and intact peptides were detected in the Orbitrap at a resolution of 70,000. Peptides were then selected for MS/MS using the NCE setting as 28, and the fragments were detected in the Orbitrap at a resolution of 17,500. A data-dependent procedure alternated between one MS scan followed by 20 MS/MS scans with 15.0 s dynamic exclusion. Automatic gain control (AGC) was set at 5E4. Fixed first mass was set as 100 m/z. The resulting MS/MS data were processed using the Maxquant search engine (v.1.5.2.8). Tandem mass spectra were searched on the human UniProt database concatenated with the reverse decoy database. Trypsin/P was specified as the cleavage enzyme allowing up to 4 missing cleavages. The mass tolerance for precursor ions was set at 20 ppm in the first search and 5 ppm in the main search, and the mass tolerance for fragment ions was set at 0.02 Da. Carbamidomethyl on Cys was specified as fixed modification, and acetylation modification and oxidation on Met were specified as variable modifications. FDR was adjusted to $< 1\%$ and the minimum score for modified peptides was set at > 40.

Western blot analysis

Total protein lysates extracted from samples were separated with 10% sodium dodecyl sulfate-polyacryl-amide gels and transferred to a polyvinylidene fluoride membrane. The membrane was blocked with 3% BSA, followed by incubation with antibodies. The membrane was then incubated with secondary horseradish peroxidase-conjugated goat anti-rabbit or anti-mouse antibodies (Jackson ImmunoResearch Laboratories Inc., West

Grove, PA), and visualized using ImmobilonTM Western Chemiluminescent HRP substrate (Millipore). β-Actin or GAPDH was used as the loading control. The following primary antibodies were used: rabbit polyclonal anti-human PLK4, rabbit monoclonal anti-human PI3KR, AKT, PDK1, FOXO4, MCM2, MCM3, CDK2, CyclinA1+A2, CDK4, FOXO3A, CyclinB1, CyclinE1, NMHC-IIA, NMHC-IIB, HSC70, FUS, ILF3, FUBP1, DDX17, KHSRP, Bmi1, ABCG2 (#ab71394, 191606, #ab32505; 202468, 128908, 108935, 128923, 32147, 185619, 199728, 53287, 32033, 33911, 138498, 230823, 51052, 124923, 92355, 181111, 180190, 140648, 126783, 108312, Abcam, Cambridge, MA), rabbit monoclonal anti-human PLK1, MDR1, Nanog, p308AKT, pS253FOXO3a, pS1943NMHC-II-A, YAP, pS109YAP, pS127YAP, pS397YAP, TAZ, YAP/TAZ, pS89TAZ, rabbit polyclonal anti-human p473AKT, pS294FOXO3a, pFOXO4, pFOXO1, KLF4，NMHC-IIC (4513, 13342, 4903, 2965, 13129, 14611, 14074, 53749, 13008, 13619, 70148, 8418, 59971, 9271, 5538, 9471, 9461, 4038, 3405; Cell Signaling Technology, Beverly, MA), and rabbit polyclonal anti-human pS61YAP (GTX133953, GeneTex).

Rho activation assay

A RhoA activation assay kit (Cytoskeleton, BK036) was used to detect the quantity of GTP-RhoA. After lysis, CRC cells were centrifuged at 13000g for 10 minutes at 4°C. The supernatants were utilized for the subsequent experiment. Ten percent of the supernatant was combined with loading buffer, and the remaining supernatant was combined with beads conjugated with the Rho-binding domain of rhotekin for two hours at 4°C. Following two rounds of washing, the beads were eluted using a Western Blot loading buffer.

Immunofluorescence staining

To detect the effect of LS-1-2 on the nuclear translocation of the transcription factor FOXO3, the CRC cells were seeded at a density of 15,000 cells per well in 96-well plates and incubated overnight. The cells were then serum starved for 24 h with the serum-free medium F12 followed by fresh media change containing 10% FBS with or without 1 μM LS-1-2 and incubation for 2 h. Cells were fixed and stained with FOXO3 (ab53287, Abcam, Cambridge, MA) or phospho-FOXO3 (5538, Cell Signaling Technology, Beverly, MA) primary antibodies and FITC-goat anti-Rabbit IgG (H + L) cross-adsorbed secondary antibodies (dilution 1:20, Cat. #F-2765, Invitrogen, Carlsbad, CA, USA). Cells were also labeled with DAPI for nuclear staining. The images were acquired using a confocal microscope (Zeiss, Oberkochen, Germany). The green fluorescence indicated FOXO3 or phospho-FOXO3 expression, and the blue fluorescence indicated the nuclei.

For detecting the colocalization of LS-1-2 and NMHC-IIA in CRC, cells were treated with biotin-tagged LS-1-2 (LS-1-2–Biotin) for 12 h. Cells were fixed and stained with biotin orNMHC-IIA (ab201341, ab138498, Abcam, Cambridge, MA) primary antibodies and eFluor570-F(ab')2-goat anti-mouse IgG/IgM (H + L) (dilution 1:20, Cat. # 41-4010-82, Invitrogen) or FITC-goat anti-Rabbit IgG (H + L) cross-adsorbed secondary antibodies (dilution 1:20, Cat. #F-2765, Invitrogen, Carlsbad, CA, USA). DAPI (Beyotime, Shanghai, China) was used to stain nuclei before capturing images. The images were acquired using a confocal microscope (Zeiss, Oberkochen, Germany). The green fluorescence indicated NMHC-IIA expression, the red fluorescence indicated Biotin, and the blue fluorescence indicated the nuclei.

IHC analysis

IHC was carried out using rabbit monoclonal anti-human pS1943NMHC-IIA (14611, Cell Signaling Technology, Beverly, MA). Two skilled pathologists individually reviewed each image. The intensity of staining was used to measure the immunoreactivity of the proteins detected, and the percentage of immunoreactive cells was scored as follows: tissues that showed no staining received a score of 0, those that showed faint or moderate staining to strong staining in less than 25% of cells received a score of 1, those that showed strong staining in 25% to 50% of cells received a score of 2, and tissues that showed strong staining in more than 50% of cells received a score of 3.

Molecular docking and Molecular dynamics

The active sites of NMHC IIA were predicted using CASTp software, and the possible active site was selected as the ligand-binding domain, with the corresponding volume of the active site being 71.031 angstroms. LS-1-2 ligand was constructed using ChemDraw software. CK2A1 protein's 3D structure was obtained from the RCSB PDB data bank (http://www.rcsb.org, PDB ID:6YPG). Following the acquisition of the NMHC IIA protein and LS-1-2 ligand 3D structures, Rosetta8 was used to carry out the molecular docking procedure, which included blind docking and precise docking. We identified the final conformer with the lowest binding energy based on a maximum of 100 conformers that were derived using precise docking. The AMBER FF99SB and AMBER PARM99 force fields were utilized for protein and ligand system simulations, respectively [3]. The final average structure was derived from 3000 snapshots taken from the last three ns trajectories. The initial NMHC IIA structure and the final molecular dynamics of NMHC IIA with LS-1-2 ligand conformation were also docked with the CK2A1 kinase structure by a similar process.

Firstly, we placed the protein and the aptamer roughly to make them face each other (within ~10 Å), in which the position of all heavy atoms of protein and ligand systems were strictly restrained, and obtained at least 2000 initial docking conformations. The precise docking was performed using these initial docking conformations as input, during which the movements of the side chains of the protein residues at the binding pocket and the ligand were allowed. Based on a maximum number of 100 conformers extracted from precise docking, we obtained the final conformer owing the lowest binding energy.

For simulations of the protein and ligand systems, the AMBER FF99SB and AMBER PARM99 force fields were used respectively. Firstly, the complexes were restrained by a harmonic potential of the form k (Δx)2 where the force constant k was 100 kcal/mol-1Å-2. Then, we optimized the water molecules and counter ions using the steepest descent and conjugate gradient for 2500 steps separately. After that, we optimized the entire systems without any constraint using the first step method, following by an annealing simulation process under a weak restraint (k=100 kcal/mol-1Å-2). The complex systems were heated from 0 to 298 K gradually over 500 ps in the NVT ensemble (i.e., N represents the number of particles, V represents the volume and T represents the temperature of the systems, and their product was a constant). After this heating phase, we performed a 10 ns MD simulation under 1 atm with a constant temperature at 298 K and a constant pressure maintained by isotropic position scaling algorithm with a relaxation time of 2 ps. Based on 3000 snapshots extracted from the last 3 ns trajectory, we obtained the final average structure. The initial MYH9 structure and the final molecular dynamics MYH9 with LS1-2 ligand conformation were also docked with the CK2A1 kinase structure as the similar process.

Cellular thermal shift assay (CETSA)

For the cell lysate CETSA experiments, HCT116 cells with MYH9 overexpression (with or without mutations on phosphorylation sites S1714 and S1943) were harvested, washed, and diluted in PBS with a protease inhibitor cocktail. The cell suspensions were freeze-thawed three times using liquid nitrogen. The soluble fraction (lysate) was separated from the cell debris by centrifugation at 20,000 ×*g* for 20 min at 4 °C. The cell lysates were diluted with an appropriate buffer and divided into two aliquots, with one aliquot being treated with LS-1-2 (10 μM) and the other aliquot treated with the diluent of DMSO (control). After 1-h incubation at room temperature, the respective lysates were divided into smaller (50 μL) aliquots and heated individually at different temperatures for 3 min followed by cooling for 3 min at room temperature. The lysates were then centrifuged at 20,000 ×*g* for 20 min at 4 °C in order to separate the soluble fractions from precipitates. The supernatants were analyzed by sodium dodecyl sulfate-polyacrylamide gel electrophoresis (SDS-PAGE) followed by western blot analysis.

For the intact cell experiments, two aliquots of HCT116 cells with MYH9 overexpression were treated by 10 μM drug and diluent of DMSO (control), respectively, under 37 °C for 1 h. The cells were washed and suspended in PBS with a protease inhibitor cocktail. The cell suspension was divided into smaller (50 μL) aliquots and heated individually at different temperatures for 3 min followed by cooling for 3 min at room temperature. The heated suspensions were lysed using 2 cycles of freeze-thawing with liquid nitrogen and centrifuged at 20,000 ×*g* for 20 min at 4 °C. The soluble fractions were isolated and analyzed by western blot.

Table S1. Clinical characteristics of colorectal cancer patients of Cohort 1

Clinical features	Number
**Gender**	
Male	161
Female	133
**Age**	
Mean± SD	59.55 ± 0.7194
**Venous invasion**	
Positive	111
Negative	183
**T stage**	
T1	2
T2	27
T3	213
T4	52
**N stage**	
N0	111
N1	95
N2	88
**M stage**	
M0	134
M1(liver metastasis)	160
**TNM stage**	
I	22
Π	58
III	54
IV	160
**Differentiation**	
Mucinous adenocarcinoma	13
Poor	57
Moderate	158
Well	66
**Histological type**	
Adenocarcinoma	281
Mucinous adenocarcinoma	13

**Lymph node metastasis**	
**Positive**	**184**
**Negative**	**110**
**Adjuvant chemotherapy**	
**Yes**	**228**
**No**	**63**
**Missing data**	**3**
**Location**	
**Colon**	**177**
**Rectum**	**117**

Table S2. Clinical characteristics of colorectal cancer patients for PDXs

Case	Gender	Age	Diffferentiation	location	Histological type	Lymphovascular invasion	Lymph node metastasis	Liver metastasis	TNM stage	KRAS	Mismatch repair status
1	Female	56	Moderate	Colon	Adenocarcinoma	Positive	Negative	Negative	II	G13D	MSS
2	Male	48	Moderate	Colon	Adenocarcinoma	Positive	Positive	Positive	IV	G13D	MSI
3	Male	51	Mucinous adenocarcinoma	Rectal	Mucinous adenocarcinoma	Negative	Positive	Positive	IV	G12A	NA

Table S3. Clinical characteristics of colorectal cancer patients for PDOs used for RNA-seq

Case	Gender	Age	Diffferentiation	location	Histological type	KRAS	Mismatch repair status
PDO1	Male	56	Moderate	Colon	Adenocarcinoma	A146T	MSS
PDO2	Female	40	Moderate	Colon	Adenocarcinoma	G12D	MSS
PDO3	Male	59	Mucinous adenocarcinoma	Rectal	Mucinous adenocarcinoma	G12D	MSS
PDO4	Male	56	Mucinous adenocarcinoma	Colon	Mucinous adenocarcinoma	G12D	MSS
PDO5	Female	49	Moderate	Rectal	Adenocarcinoma	G12D	MSS
PDO6	Male	66	Low	Rectal	Adenocarcinoma	Wild	MSS
PDO7	Female	50	Moderate	Colon	Adenocarcinoma	Wild	MSS
PDO8	Female	52	Moderate	Colon	Adenocarcinoma	Wild	MSS
PDO9	Male	73	Moderate	Rectal	Adenocarcinoma	Wild	MSS
PDO10	Male	56	Moderate	Rectal	Adenocarcinoma	Wild	MSS

Table S4. the KRAS mutation status of the CRC cells used in this study

Cell line	KRAS	Cell line	KRAS
CL11	p.Q61H	LOVO	p.G13D
CL40	p.G12D	LS174T	p.G12D
LS513	p.G12D	HT29	wild-type
HCT116	p.G13D	RKO	wild-type
HCT8	p.G13D	Difi	wild-type
SW480	p.G12V	Caco-2	wild-type
SW620	p.G12V		

Table S9. Correlation between pS1943 MYH9 and clinicopathological parameters

	**pS1943 MYH9**
**Differentiation**	
Correlation coefficient	0.234
P value	<0.0001
**Primay tumor (T) stage**	
Correlation coefficient	0.003
P value	0.966
**Lymph node (N) m etastasis**	
Correlation coefficient	-0.038
P value	0.533
**Distant metastasis (M)**	
Correlation coefficient	0.200
P value	0.001
**TNM stage**	
Correlation coefficient	0.160
P value	0.009
**CD44**	
Correlation coefficient	0.225
P value	0.001
**MYH9**	
Correlation coefficient	0.234
P value	<0.0001
**Histological type**	
U	0.789
P value	0.429
**Venous invasion**	

U	0.379
P value	0.705

Spearman's rho correlation test (two tailed) was used to estimate the correlation with clinico-pathological variables.

Table S10. The sequences of siRNAs against specific targets

Gene name	Sequence
**MYH9 siRNA-1**	**GCATCAACTTTGATGTCAA**
**MYH9 siRNA-2**	**GCAACACGGAGCTGATCAA**

Figure S1

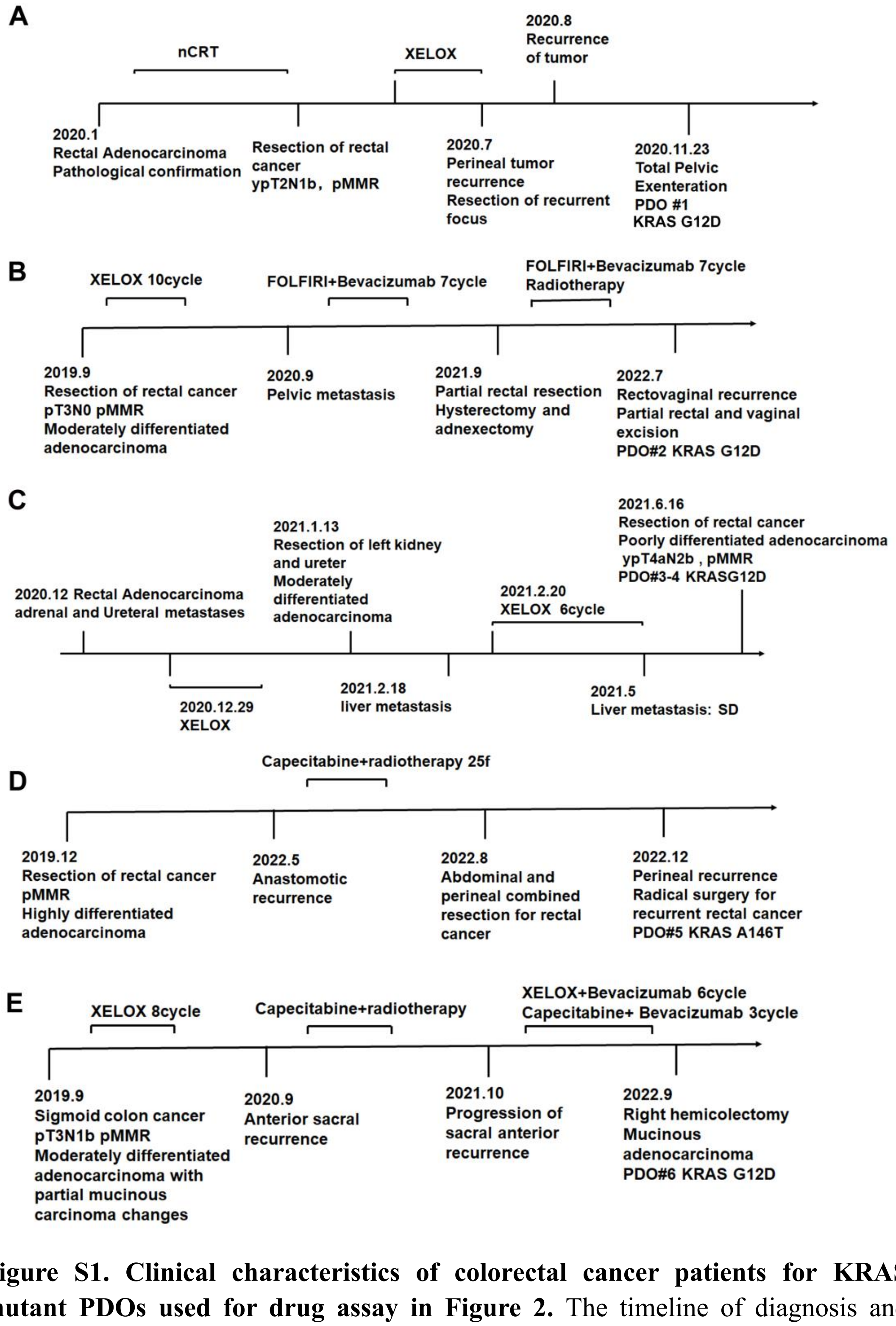

Figure S1. Clinical characteristics of colorectal cancer patients for KRAS mutant PDOs used for drug assay in Figure 2. The timeline of diagnosis and treatment procedures for patients. **A**. PDO #1. **B**. PDO #2. **C**. PDO #3-4. **D**. PDO #5. E. PDO #6.

Figure S2

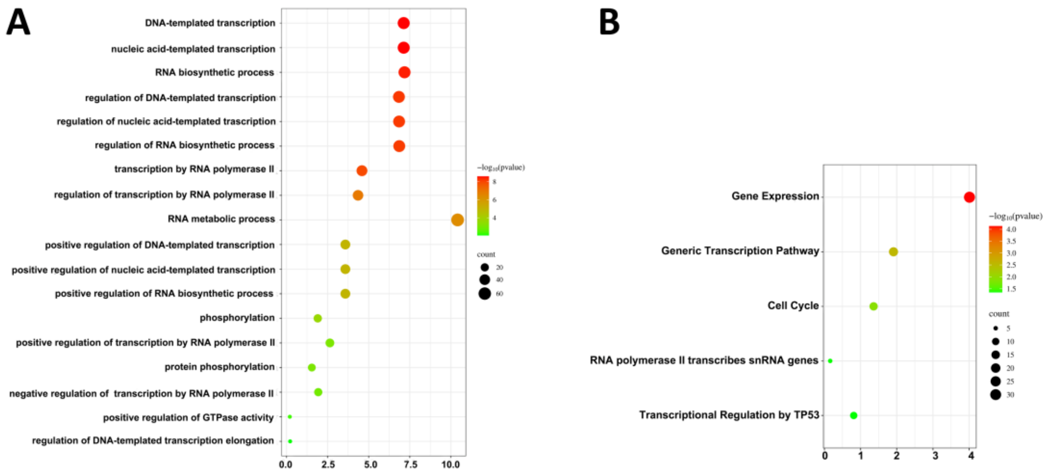

Figure S2. Pathway enrichment analysis comparing KRAS-mutant CRC epithelia cells with KRAS-wild CRC epithelia cells from a single-cell transcriptomic data. **A**. Gene Ontology (GO) enrichment analysis showing top 18 upregulated biological processes in KRAS-mutant CRC epithelia cells. **B**. KEGG pathway analysis identifying five significantly activated signaling cascades in KRAS-mutant CRC epithelia cells.

Figure S3

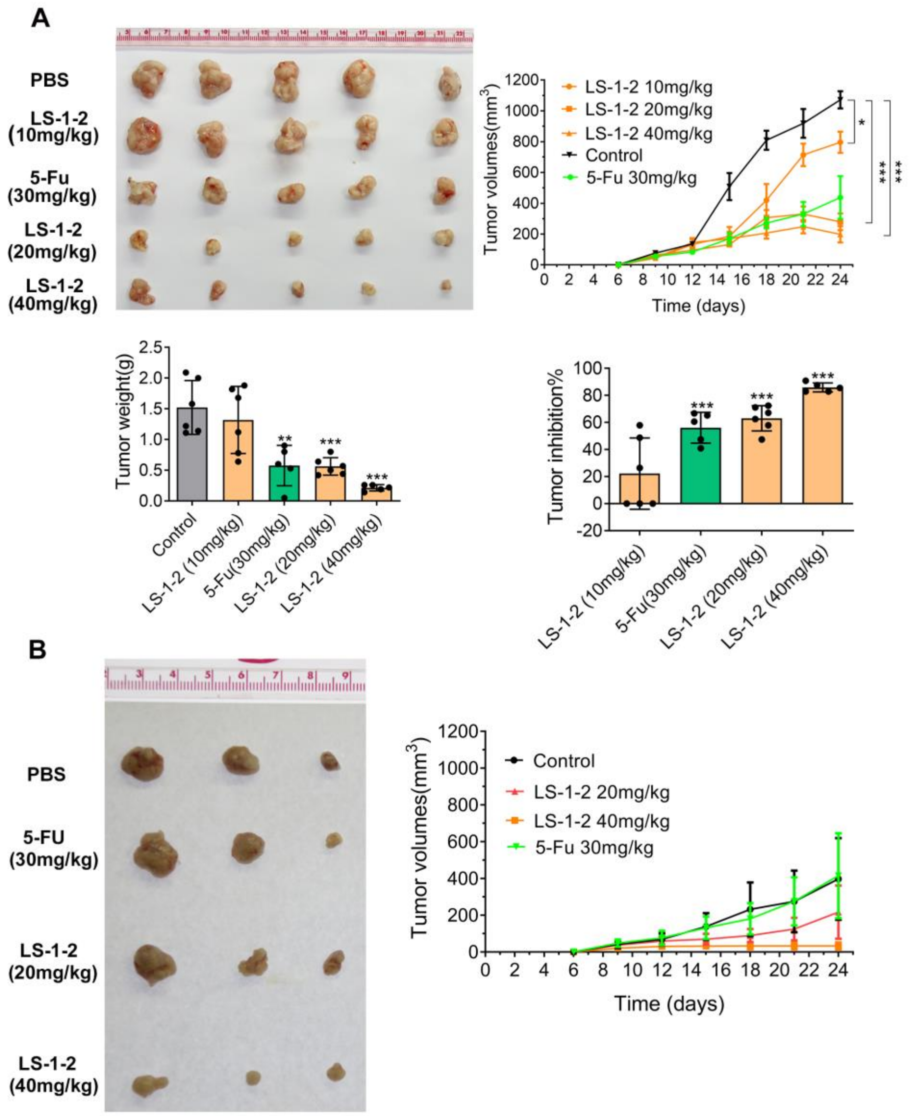

Figure S3. LS-1-2 exhibits robust anti-tumor efficacy in CRC CDX models. A. Representative image of HCT116 xenograft tumors dissected from NOD/SCID mice in different groups (upper panel). The mice in the treatment group were given PBS, LS-1-2 (10, 20, 40 mg/kg, d1–d5/week), or 5-FU (30 mg/kg, once weekly) intraperitoneal injection for 18 days. Tumor volumes and mouse weight were measured every three days. The tumor volumes are plotted as the mean ± S.E.M. of n=5 mice per group. Tumor weights and tumor inhibition rates in different groups of mice are shown. The data are presented as the mean ± S.D. of n=5 mice per group. *P* values were determined by one-way ANOVA. *, $p < 0.05$.**, $p < 0.01$.***, $p < 0.001$.**B**. Representative image of 5-FU resistant HCT8-5Fu xenograft tumors dissected from NOD/SCID mice in different groups (upper panel). The mice in the treatment group were given PBS, LS-1-2 (20, 40 mg/kg, d1–d5/week), or 5-FU (30 mg/kg, once weekly) intraperitoneal injection for 18 days. The tumor volumes are plotted as the mean ± S.E.M. of n=3 mice per group.

Figure S4

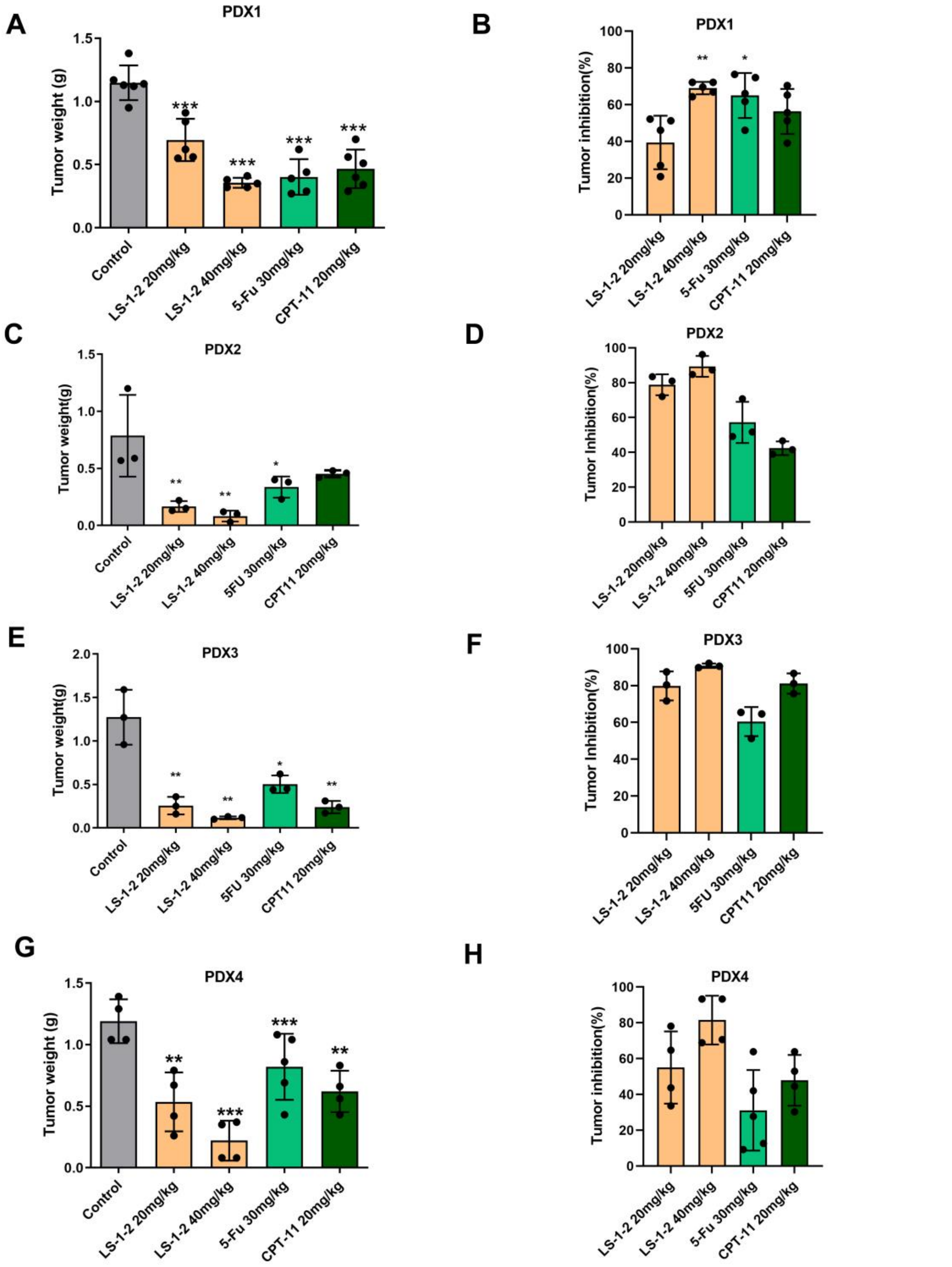

Figure S4. The effect of LS-1-2 on tumor weight and tumor inhibition rate in CRC PDX models. The data are presented as the mean ± S.D. of n=5 mice per group. *P* values were determined by one-way ANOVA. *, $p < 0.05$.**, $p < 0.01$.***, $p < 0.001$.

Figure S5

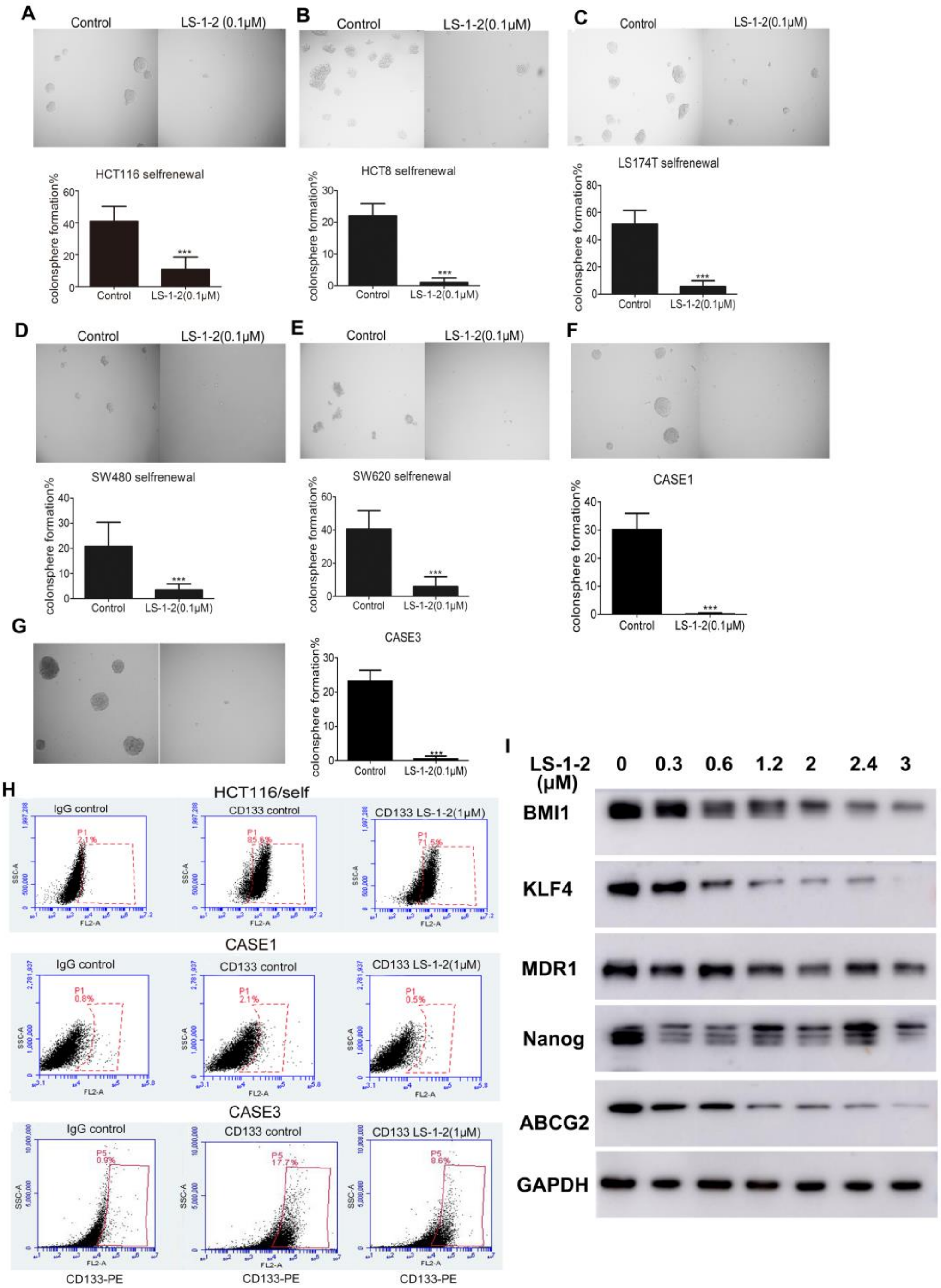

Figure S5. LS-1-2 prevents colonosphere formation and depletes colorectal CSC markers *in vitro*. A-G. LS-1-2 prevents colonosphere formation. HCT116, HCT8, LS174T, SW480, SW620, and two PDC cells (CASE1 and CASE3) (100 cells/well) were plated for colonosphere formation in the presence of DMSO or 0.1 μmol/L LS-1-2. Colonospheres (> 60 mm) were counted at 2 weeks. Scale bars, 50 mm. Data are presented as the mean of three independent experiments ± SE (n=5). Student's *t*-test, ***, $p < 0.001$. **H.** LS-1-2 depletes colorectal CSC markers *in vitro*. LS-1-2 depletes CD133+ cells. HCT116/self cells and two PDC cells (CASE1 and

CASE3) were treated for 24 h with DMSO or 1 μM LS-1-2, and CD133+ cells were detected by flow cytometry. **I.** The effects of LS-1-2 on several cancer stem cell markers in HCT116/self cells using western blotting. HCT116/self cells were treated with DMSO or indicated doses of LS-1-2. Data refer to a representative result from three repeats.

Figure S6

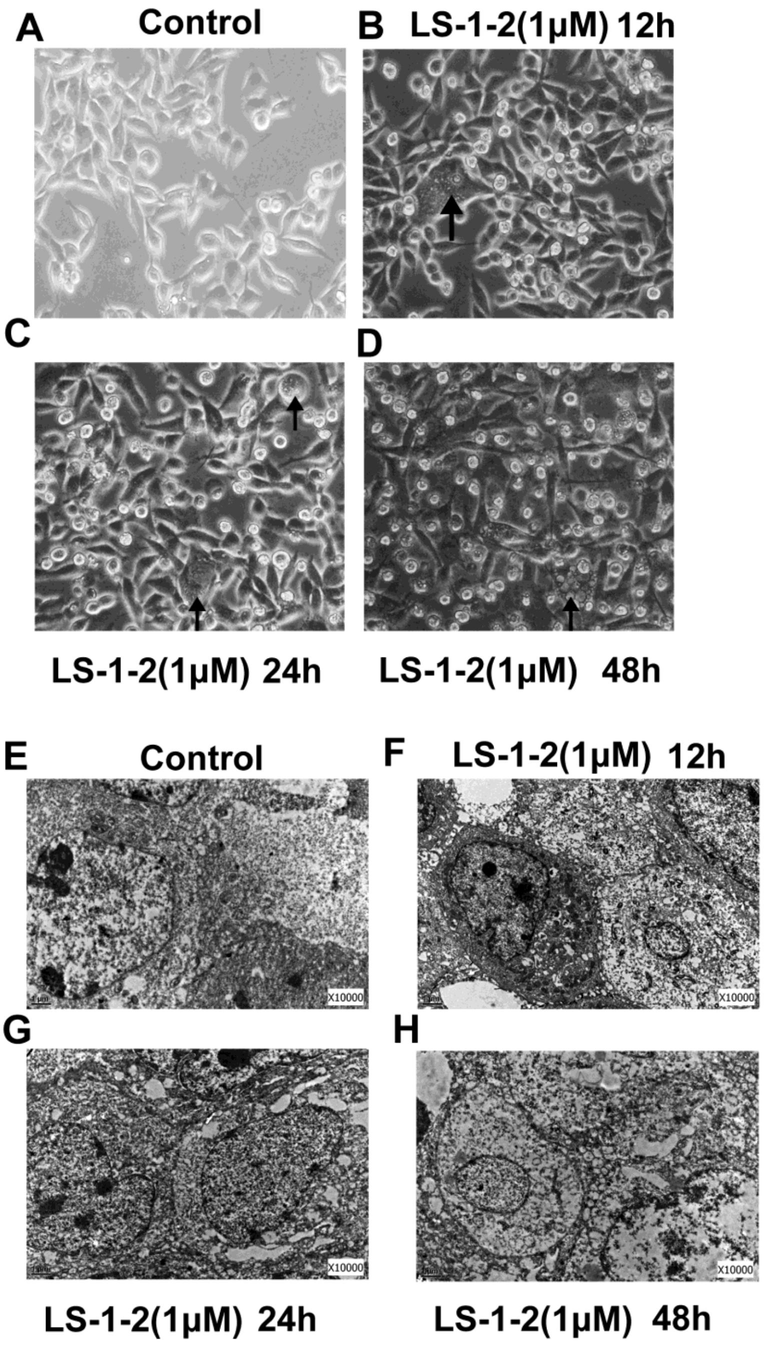

Figure S6. The influence of morphology produced in HCT116 cells with LS-1-2 treatment by light microscope(A-D) and transmission electron microscope (E-H).

Figure S7

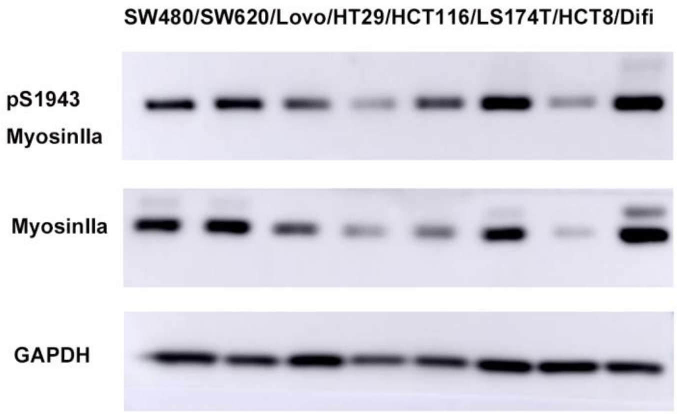

Figure S7. The expression of myosin IIa and pS1943myosin IIa in CRC cells.

Figure S8

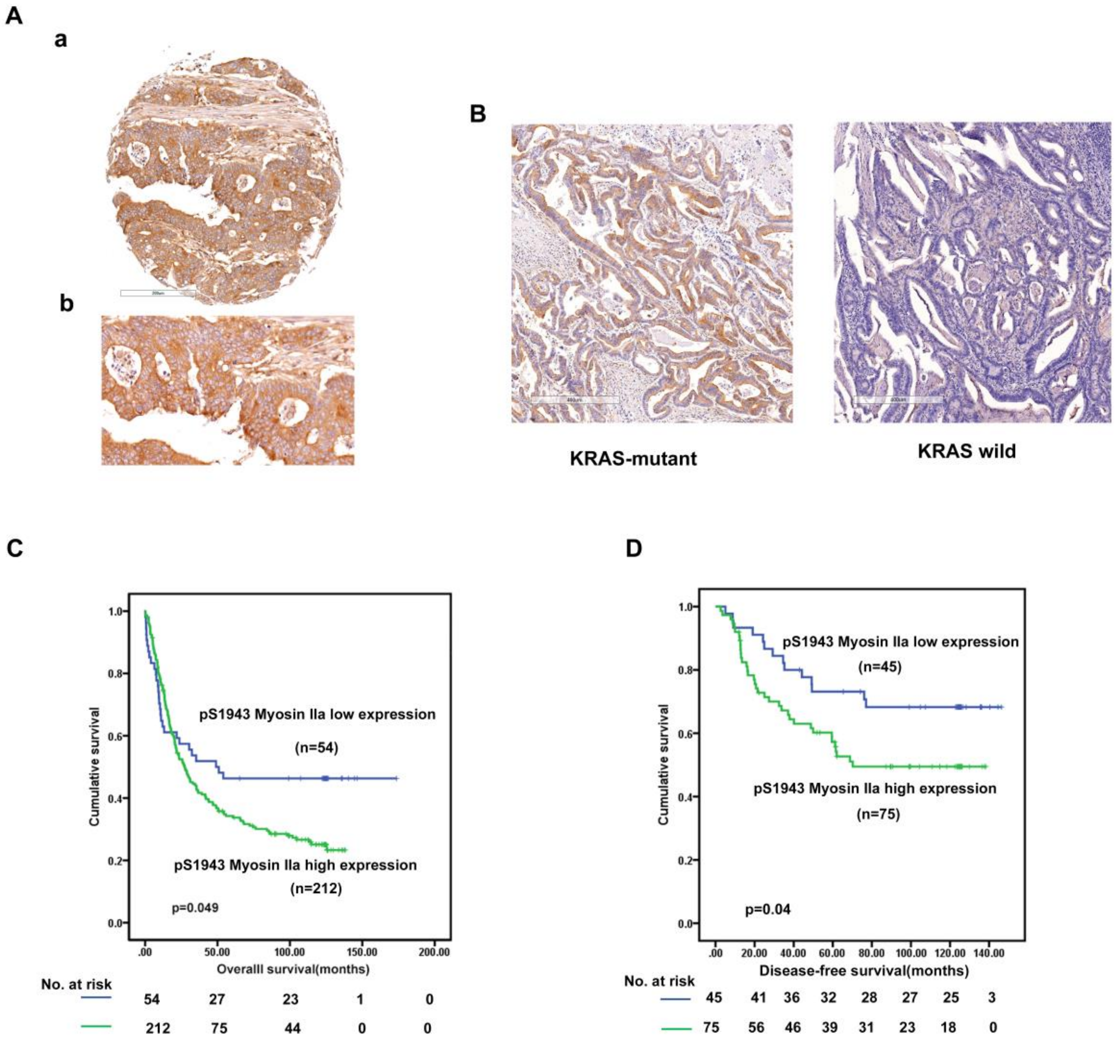

Figure S8. Association between pS1943 Myosin IIa expression and prognosis with colorectal cancer using IHC (cohort 1). A. Expression of pS1943 Myosin IIa in the indicated colorectal cancer tissues using IHC analysis. Positive staining is shown in brown. **B.** The expression of pS1943 Myosin IIa on KRAS-mutant and KRAS-wild CRC. **C.** Kaplan–Meier analysis of the correlation between pS1943 Myosin IIa expression and OS in CRC patients. **D.** Kaplan–Meier analysis of the correlation between pS1943 Myosin IIa expression and DFS in CRC patients. Positive staining is shown in brown. The number of patients per group indicated on graphs. Using the log-rank test, statistical significance was set at $p < 0.05$.

Figure S9

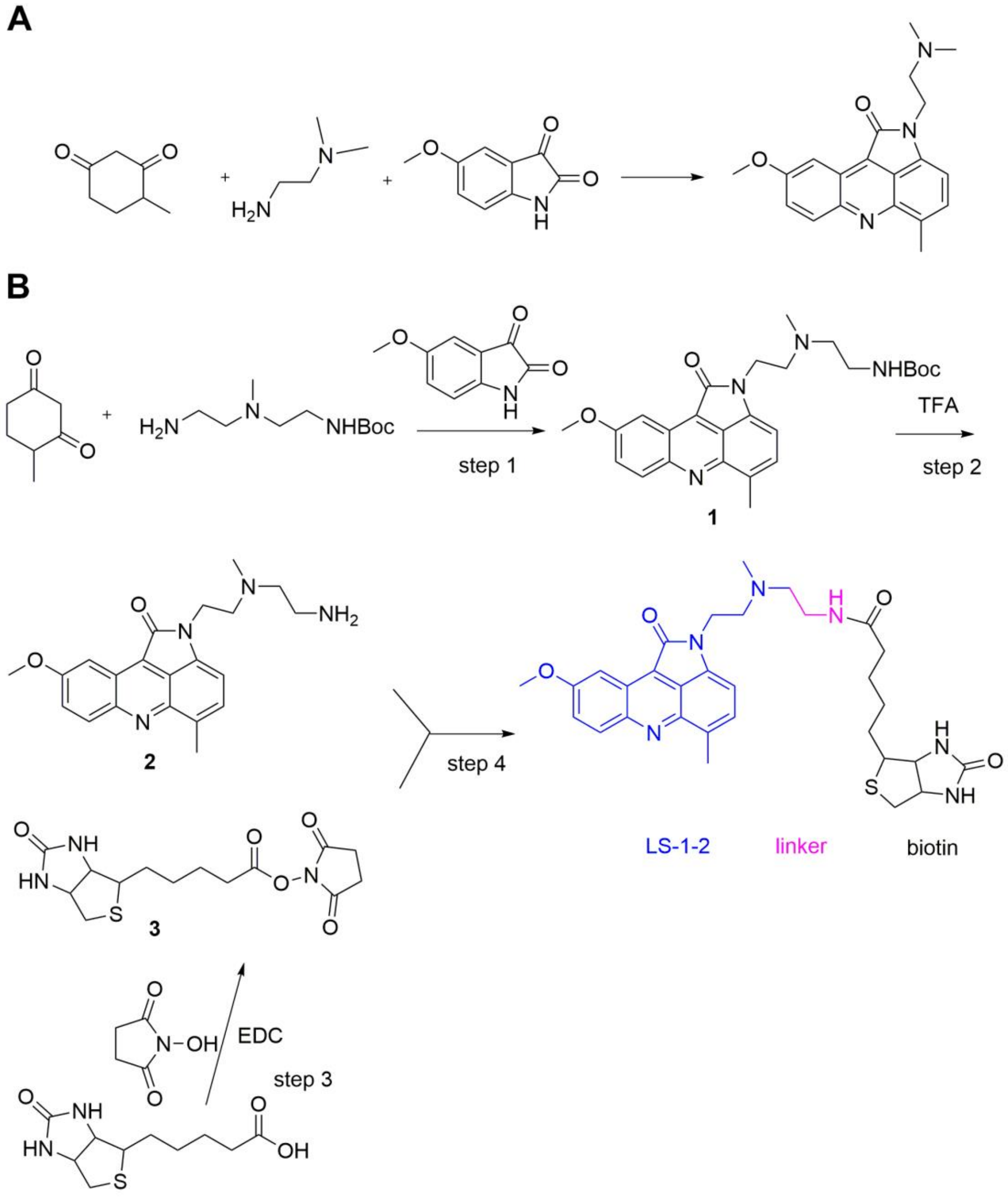

Figure S9. Synthesis of LS-1-2(A) and synthesis of biotin-LS-1-2(B).

Figure S10

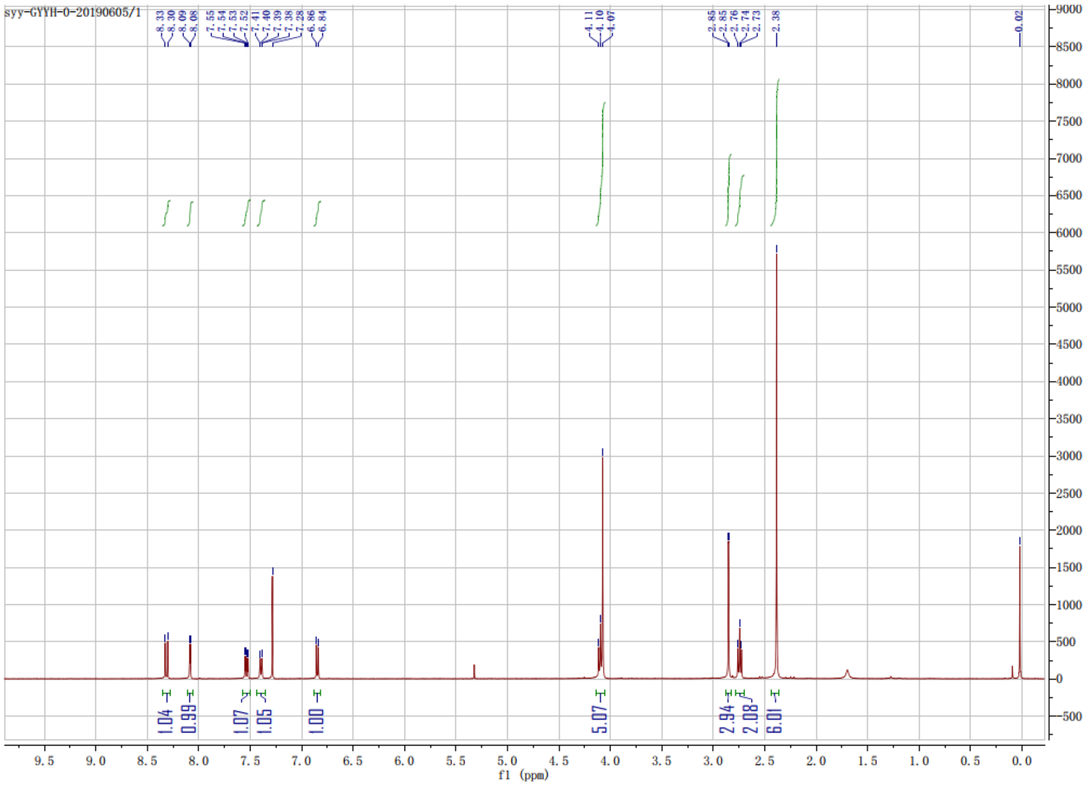

Figure S10. ^{1}H NMR of LS-1-2

Figure S11

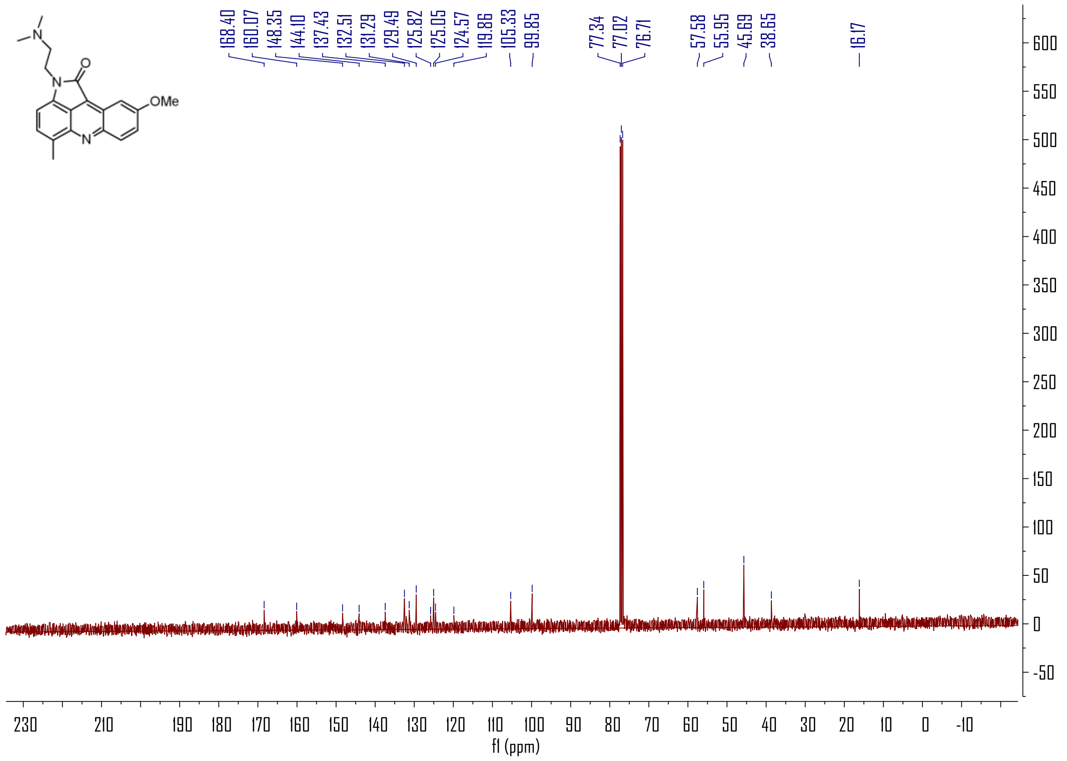

Figure S11. ^{13}C NMR of LS-1-2

Figure S12

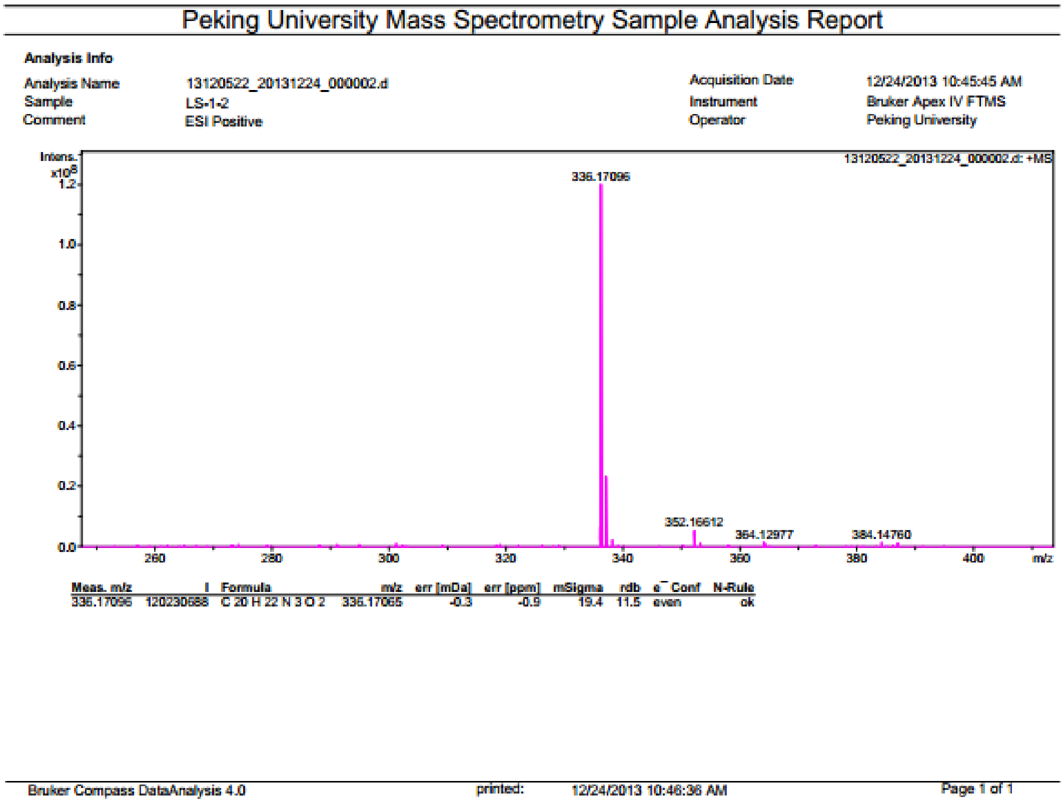

Figure S12. MS of LS-1-2

References:

[1] L.Z. Meng X, Liu S, Yu S, Que L, Fused acridine derivative and pharmaceutical composition, preparation method and use thereof, 2015-2-15.

[2] Y. Kimura, S. Ito, Y. Shimizu, M. Kanai, Catalytic anomeric aminoalkynylation of unprotected aldoses, Org Lett, 15 (2013) 4130-4133.

[3] H. Gouda, I.D. Kuntz, D.A. Case, P.A. Kollman, Free energy calculations for theophylline binding to an RNA aptamer: Comparison of MM-PBSA and thermodynamic integration methods, Biopolymers, 68 (2003) 16-34.
